# Exploring the biological functions and immune regulatory roles of IRAK3, TNFRSF1A, CX3CR1, and JUNB in T2DM combined with MAFLD: integrated bioinformatics and single-cell analysis

**DOI:** 10.3389/fimmu.2025.1587225

**Published:** 2025-08-22

**Authors:** Qin Wang, Xiaoqi Li, Kaidierdan Wushoulaji, Jinyang Wang, Li Wan, Ye Yang, Xueli Gong

**Affiliations:** ^1^ Department of Geriatric Integrative, The Second Affiliated Hospital of Xinjiang Medical University, Xinjiang Medical University, Urumqi, Xinjiang, China; ^2^ The Second Affiliated Hospital of Xinjiang Medical University, The Second Clinical Medical College, Urumqi, Xinjiang, China; ^3^ Department of Clinical Medicine, Xinjiang Medical University, Urumqi, Xinjiang, China; ^4^ Department of Gynecology, The Fourth Clinical Medical College of Xinjiang Medical University, Urumqi, Xinjiang, China; ^5^ Department of Pathophysiology, School of Basic Medical Science, Xinjiang Medical University, Urumqi, Xinjiang, China

**Keywords:** T2DM, MAFLD, single-cell RNA sequencing, biomarkers, immune signaling pathway

## Abstract

**Objective:**

Diabetes mellitus combined with nonalcoholic fatty liver disease is a prevalent and intricate metabolic disorder that presents a significant global health challenge, imposing economic and emotional burdens on society and families. An in-depth understanding of the disease pathogenesis is crucial for enhancing diagnostic and therapeutic efficacy. Therefore, the study aims to identify and validate autophagy-related diagnostic biomarkers associated with T2DM-associated MAFLD, investigate regulatory mechanisms in disease progression, and explore cellular diversity within the same tissue using single-cell sequencing data.

**Methods:**

This study utilized four datasets retrieved from the Gene Expression Omnibus (GEO) database: GSE15653, GSE89632, GSE24807 and GSE23343. The analysis involved variance analysis, WGCNA analysis, PPI network construction, machine learning application, examination of autophagy-related gene sets, and diagnostic ROC analysis to identify and validate autophagy-related biomarkers in T2DM combined with MAFLD within an independent external dataset. Functional enrichment analysis, immune infiltration analysis, and validation of gene significance in T2DM combined with MAFLD progression were conducted using animal experiments to understand the biological functions and immunomodulatory roles of key biomarkers. Cellular diversity within liver tissues was characterized at the single-cell level, exploring interrelationships, differentiation, and developmental trajectories among cell populations through cellular communication and pseudo-temporal analyses.

**Results:**

The study identified four key biomarkers (IRAK3, TNFRSF1A, CX3CR1, JUNB). Real-time fluorescence quantitative PCR analysis in animal experiments demonstrated significantly higher mRNA expression levels of IRAK3, TNFRSF1A, CX3CR1, and JUNB in T2DM and MAFLD rat liver tissues compared to the control group. Quantitative immunohistochemical analysis revealed notably elevated protein expression levels of IRAK3, TNFRSF1A, CX3CR1, and JUNB in liver tissues of rats with T2DM and MAFLD when contrasted with the control group (P < 0.05). Enrichment analysis indicated associations of T2DM combined with MAFLD pathogenesis with pathways such as the NF-kappa B signaling pathway, MAPK signaling pathway, Fluid shear stress and atherosclerosis, Insulin resistance, and Cytokine-cytokine receptor interaction. Correlative analysis uncovered connections between immune infiltration and the identified genes. Single-cell transcriptomic analysis highlighted the differentiation of CX3CR1, JUNB, and TFRC in various single-cell-annotated populations. The pseudo-temporal analysis of epithelial cells identified enriched genes at crucial nodes related to “Leukocyte transendothelial migration”, “Lipid and atherosclerosis”, and “Type II diabetes mellitus” signaling pathways. Additionally, four cellular communication signaling pathways (TNF, CXCL, VEGF, and MIF) potentially significant in T2DM combined with MAFLD progression were identified through cell communication analysis.

**Conclusion:**

This study unveiled potential associations and key biomarkers (IRAK3, TNFRSF1A, CX3CR1, JUNB) concerning T2DM combined with MAFLD and relevant pathways, offering novel insights for the investigation of these two conditions.

## 1 Introduction

Type 2 Diabetes Mellitus (T2DM) is a prevalent metabolic disorder characterized by insulin resistance and relative insulin deficiency, resulting in hyperglycemia. The International Diabetes Federation reported approximately 537 million adults living with diabetes globally in 2021, with T2DM representing about 90% of these cases (1). Metabolic dysfunction-associated fatty liver disease (MAFLD), formerly termed non-alcoholic fatty liver disease (NAFLD), is characterized by excessive fat accumulation in the liver and affects nearly 25% of the global population. The surge in MAFLD cases mirrors shifts in the environment and lifestyle driven by rapid global industrialization; environmental elements like air pollution may heighten MAFLD risk, compounding socio-economic challenges. In essence, MAFLD represents an escalating global health issue necessitating robust preventive and therapeutic strategies. The pathogenesis of MAFLD is multifactorial, heavily influenced by obesity, insulin resistance, and dyslipidemia. The co-occurrence of MAFLD in T2DM patients raises concerns as it increases the risk of liver-related complications and cardiovascular events (2, 3). This relationship is intricate, with overlapping metabolic pathways and inflammatory processes that may drive disease progression. Furthermore, MAFLD is closely linked to metabolic dysregulation, primarily insulin resistance, which exacerbates liver fat accumulation and inflammation (4). The interplay between T2DM and MAFLD is significant; both conditions share common risk factors and pathophysiological mechanisms, including obesity and dyslipidemia, creating a vicious cycle that further deteriorates metabolic health (5). Given the insidious progression of T2DM with MAFLD, identification of novel diagnostic markers significantly impacts the early detection and treatment such kind of disease.

The correlation between autophagy and MAFLD has garnered substantial attention. Autophagy, a conserved catabolic process, plays a crucial role in physiological functions by degrading misfolded proteins, eliminating dysfunctional organelles, and influencing growth and aging. Autophagy’s formation of autophagosomes, which ensnare substances for degradation and unite with lysosomes housing hydrolytic enzymes, results in the breakdown of encapsulated components into biomolecule monomers like amino acids, fatty acids, and nucleotides for cellular recycling. An integral mechanism of MAFLD involves diminished autophagic capacity; the accumulation of excess lipids in hepatocytes can hinder the autophagic process, impeding efficient lipid removal and exacerbating fatty liver progression. Moreover, oxidative stress emerges as a significant determinant of autophagic levels in MAFLD; augmented reactive oxygen species (ROS) production can disrupt autophagy regulation and functionality. Research indicates a prevalent magnesium deficiency in individuals with obesity and metabolic syndrome. Magnesium supplementation demonstrates effectiveness in ameliorating metabolic disorders such as obesity and fatty liver by modulating cellular lipid metabolism through autophagy stimulation via the AMPK/mTOR pathway, thereby curbing lipid buildup. Recognizing autophagy’s pivotal role in MAFLD pathogenesis, drug development targeting the autophagic process holds promise as a novel therapeutic avenue for MAFLD treatment.

The interplay between T2DM and MAFLD has not been fully elucidated, particularly regarding the role of autophagy-related genes. To address this knowledge gap, our study aimed to examine the relationship between autophagy-related genes and endoplasmic reticulum stress-associated genes in the context of T2DM and MAFLD. We utilized relevant datasets from the GEO database for differential analysis to identify differentially expressed genes (DEGs). Subsequently, we employed WGCNA, PPI network analysis, machine learning techniques, diagnostic ROC analyses, and enrichment assessments to identify key biological markers and elucidate their pathways, gaining deeper insights into the underlying biological mechanisms.

In our rat model of T2DM combined with MAFLD, we verified the expression levels of predicted genes, identifying IRAK3, TNFRSF1A, CX3CR1, and JUNB as potentially significant candidates. Further, leveraging single-cell analysis, we elucidated potential mechanisms underlying the progression of T2DM and MAFLD, focusing on precise cellular localization, differentiation, developmental trajectories within pertinent cell populations, and potential intercellular communication networks. This study not only enhances our understanding of the pathogenesis of T2DM combined with MAFLD but also identifies diagnostic factors linked to autophagy. Such advancements are vital for improving early identification, precise diagnosis, personalized treatment, and disease monitoring for these conditions. The findings provide novel opportunities and challenges for the prevention and treatment of T2DM and MAFLD. The flow diagram of this study is showed in the **Supplementary Figure 1**.

## 2 Material and methods

### 2.1 Identification of differential expression genes

The datasets GSE15653, GSE89632, GSE24807 and GSE23343 were obtained from the GEO database through the GEOquery package (version 2.72.0) (6),please refer to **Supplementary Table S1** for more details.GSE15653 and GSE89632 served as training sets, while GSE24807 and GSE23343 was utilized as the validation set. The MAFLD samples within the GSE89632 dataset were categorized by different stages: Simple Steatosis (SS) and Non-Alcoholic Steatohepatitis (NASH), and were designated as GSE89632_Simple Steatosis (GSE89632_SS) and GSE89632_Non-Alcoholic Steatohepatitis (GSE89632_NASH), respectively. The data from GSE15653, GSE89632_SS, and GSE89632_NASH were normalized using the normalizeBetweenArrays function from the limma package (7). Principal component analysis (PCA) was performed on the normalized dataset. Difference analysis was performed separately to obtain the respective DEGs, using limma package (version 3.58.1), with screening criteria: |log2FC| > 0.5 and P < 0.05 (8, 9). Subsequent analyses included the generation of PCA plots, volcano plots, and basic numerical heatmaps using “ggplot2,” alongside the creation of complex numerical heatmaps utilizing the “ComplexHeatmap” package (version 2.20.0) (10).

### 2.2 WGCNA analysis

Weighted gene co-expression networks were analyzed using the WGCNA package (version 1.72-5) (11) for the datasets GSE89632_SS and GSE89632_NASH. Initially, the analysis focused on the top 25% of genes based on expression variance, identifying and excluding potential outlier samples through clustering analysis. Next, the pickSoftThreshold function was employed to ascertain the optimal soft threshold. After establishing the threshold, the cutreeDynamic function identified dynamic modules and set the minimum gene count required for each module (12). Initial modules were identified through the Dynamic Tree Cut (DTC) method, with similar modules subsequently merged based on the clustering relationships of modular eigengene (ME). Subsequently, the TOMsimilarity function calculated the topological molecular similarity matrix among genes, with 1,000 genes randomly selected for heatmap visualization. Pearson correlation coefficients were computed to evaluate associations between modules and clinical traits, with statistical significance assessed via the corPvalueStudent function (WGCNA R package). Modules demonstrating significant correlations with both SS and NASH (p < 0.05) were selected for downstream analysis. KEGG pathway enrichment analysis was then systematically performed on the genes within these modules.

### 2.3 Protein-protein interaction network

The results of DEGs of GSE15653 were intersected with DEGs of GSE89632_SS, GSE89632_NASH, and WGCNA modular genes, respectively, and the results were visualized in an “UpSet plot”. In order to explore the potential interactions of the above genes, we used the STRING database (https://string-db.org/) for the analysis (13), and the results were imported into Cytoscape (version 3.10.2) for the analysis of protein interaction networks (14). Meanwhile, the Maximal Clique Centrality (MCC), Degree and Molecular Network Centrality (MNC) algorithms in CytoHubba plug-in were applied to select the top 15 genes at key positions in the PPI network (15). Subsequently, the results of the three algorithms were intersected and visualized using a Wayne diagram. Chromosomal localization analysis of the intersection hub genes was performed using the “circlize package”. Finally, the correlation analysis was performed using Spearman, and the correlation pie charts were used to show the degree of association and interactions between genes, which were visualized using the “ggplot2 package”.

### 2.4 Acquisition of autophagy-related genes

In order to deeply investigate the molecular mechanisms related to autophagy in T2DM combined with MAFLD, we combined the resources from multiple databases. We obtained the autophagy-associated molecular mechanisms from PubMedGene database (https://www.ncbi.nlm.nih.gov/gene/), Genecard database (https://www.genecards.org/), and GSEA database (https://www.gsea-msigdb.org/), autophagy-related websites (http://www.autophagy.lu/), (http://hamdb.scbdd.com/home/index/) to obtain autophagy-related genes.

### 2.5 Machine learning

The GSE89632_SS and GSE89632NASH datasets underwent individual screenings using LASSO, RFE-SVM, and Boruta methods, respectively. The diagnostic LASSO coefficient screening leveraged the glmnet package (Version 4.1.7) to analyze the cleaned data, derive variable lambda, likelihood, and L1 regularization values, along with visualization. We employed ten-fold cross-validation (seed number: 2022) for validation (16). Additionally, we utilized two feature selection strategies: Recursive Feature Elimination (RFE) and the Boruta algorithm. RFE eliminates features without significant impact on Accuracy during model training repetitions (17), while the Boruta algorithm selects or eliminates significant and non-significant features in each base learner training iteration based on shadow features and Z-scores (18). This study integrated SVM base learner scores from the e1071 package (Version 1.7-14) with the RFE strategy (17) through 10*10 fold cross-validation and applied the Boruta algorithm using the Boruta package (Version 8.0.0) (18). Subsequently, the outcomes of Lasso, Boruta, and RFE-SVM screenings, combined with autophagy-related genes, were intersected to identify diagnostic biomarkers linked to autophagy in T2DM and NAFLD. These results were visually represented in Wayne diagrams.

### 2.6 Receiver operating characteristic analysis

In datasets GSE89632_SS, GSE89632_NASH and GSE15653, ROC curve analysis was performed on each of the three datasets using the pROC software package to determine the sensitivity and specificity of the above genes (19), to predict the ROC-related information and data of the variables at their respective cut-off values, and to assess the diagnostic accuracy, and the results were presented as ROC The results were quantified by area under the curve (AUC), and the AUC ranges between 0.5 and 1, and values closer to 1 indicate superior diagnostic performance of the variable in predicting clinical outcomes and visualized using ggplot2.

### 2.7 Enrichment analysis

To investigate the functions and pathways of the above biomarkers, we used the DAVID online database (https://david.ncifcrf.gov/) for GO and KEGG enrichment analysis (20–22). The “org.Hs.eg.db” package was used to convert the ID of the input gene list, and the “clusterProfiler package” was used for enrichment analysis (23). The significance of the enrichment results was assessed by calculating the z-score value for each enriched entry using the “GOplot” package (24). The results were screened for significance and biological significance at P<0.05 and FDR<0.2 (25). At the same time, GSEA(Gene Set Enrichment Analysis) was performed (26), and the results were screened according to the following criteria: normalized enrichment score |NES| > 1, FDR < 0.25, p.adjust < 0.05. Finally, the results were visualized using the “ggplot2 package”.

### 2.8 Immune cell infiltration analysis

In order to explore the immune cell infiltration in liver tissues of patients with T2DM combined with MAFLD, the “Single Sample Gene Set Enrichment Analysis” (ssGSEA) method was used in this study (27). The ssGSEA algorithm provided by the GSVA package was utilized for this purpose. Using markers specific to each class of immune cells (28) as gene sets, the enrichment score for each class of immune cells in each sample was calculated to assess the infiltration of immune cells in each sample. All analyses and visualizations were performed in R 4.2.1. We used the ggplot2 package to draw histograms to visualize the differences in immune cell infiltration between the normal and control groups. In addition, Spearman statistics were used to analyze the correlation between them one by one, and the linkET package was used for the calculation of the data portion of the network graph. The analysis results were visualized by ggplot2 package for group comparison plots, lollipop plots, correlation scatter plots, and correlation network heatmaps, thus demonstrating more intuitively the infiltration of immune cells in liver tissues of patients with T2DM combined with MAFLD.

### 2.9 Preparation of T2DM combined with MAFLD rat model

Thirty healthy clean-grade male Sprague–Dawley rats, aged 6–8 weeks, weighing 180–220 g, were provided by Wuhan Yunkron Technology Company. The animal production and use license number is DCXR(E)2018-0021. SYXK (E) 2013-0069, respectively. After 1 week of normal feeding, high-fat and high-sugar diets and regular diets were prepared according to the formula. Twenty rats were fed with high-fat and high-sugar diet, high-fat and high-sugar diet feed formula: 10% lard, 20% sucrose, 2% cholesterol, 60% ordinary feed, 8% egg yolk powder(Jiangsu Xietong Pharmaceutical Bio-engineering Co., Ltd.) and 10 rats in normal control group were fed with normal diet. Twenty rats were fed a 100 g high-fat and high-sugar diet daily with free access to food and water. Corn oil 5 mL/kg was given by gavage on an empty stomach at 8:00 am every morning. At the end of the 12th week, 10 rats in the model group were given intraperitoneal injection of streptozotocin (STZ) (30 mg/kg) overnight fasting, and the other 10 rats in the model group were not given STZ injection. Ten control rats fed a regular diet were injected with the corresponding volume of citrate buffer. Blood samples were collected from the tail vein of 10 rats fed a high-fat and high-sugar diet and injected with STZ 3 days later, and random blood glucose was≥16.7 mmol/L. Blood glucose was monitored after 2 weeks, and fasting blood glucose was detected on the 2nd, 4th, 6th, 8th, 10th, 12th and 14th days. At the 14th week, 4 rats in each group were randomly selected to complete liver ultrasound, and fatty liver results were formed to determine the success of modeling. Finally, the rats were divided into 3 groups: (1) T2DM+MASLD group (n=10); (2) High-fat and high-glucose group (n=10); (3) normal control group (n=10); After 15 weeks, the rats were sacrificed under anesthesia, and the livers were collected, rinsed with normal saline at 4°C, weighed and placed in preservation solution and stored in a refrigerator at −80°C.

### 2.10 Quantitative real time PCR

Real-time fluorescence quantitative PCR (Polymerase Chain Reaction): 0.15 g of rat liver tissue was taken from each group, and total RNA was extracted by the Trizol method. cDNA was reverse-transcribed into the corresponding cDNA in accordance with the reverse-transcription kit, and then subjected to real time Polymerase Chain Reaction (PCR)、RELB、S100A9 and SOCS1 primer sequences are shown in **Supplementary Table S2**, Chain Reaction), PCR reaction total system 20μL, PCR amplification conditions: denaturation at 95°C for 10 min, annealing at 60°C for 1 min, extension at 95°C for 15 s, 40 cycles, each sample set up 3 replicate wells, internal reference GAPDH. The results of the experiments were analyzed by Bio-Rad Fluorescence Quantitative Analysis. The results of the experiments were read by Bio-Rad fluorescence quantitative analysis software, and the quantitative calculation of the mRNA expression levels of and in each group was expressed by 2−ΔΔCt (CT is the number of cycles). The main observation indexes were RELB, S100A9, SOCS1 mRNA expression levels in rat liver tissues in each group.

### 2.11 Immunohistochemical staining

Kidney tissue specimens were fixed for 48h and paraffin-embedded. The paraffin-embedded tissue sections were serial sectioned at 3 μm, dewaxed and hydrated. 3% H 202 was incubated at room temperature for 10 min to remove endogenous peroxidase. After 10 min of citrate antigen repair, primary antibodies RELB (1:50), S100A9(1:300), SOCS1 (1:300), were added dropwise and incubated at 37°C for 25 min, and then freshly prepared DAB color development solution was added dropwise. The film was stained with hematoxylin and sealed with neutral gum. At the same time, PBS was used as negative control instead of primary antibodies. The immunohistochemical pathology pictures were analyzed using ImageScope software as follows: three different 400x fields of view were intercepted from each section, enter the analysis module of lmageScope software, and set all the dark brown color on the tissue sections as strong positive, brownish yellow as moderate positive, light yellow as weak positive, and blue nuclei as negative. Each tissue point was then identified and analyzed to find out the area (in pixels) of strong positive, moderate positive, weak positive and negative, the percentage of positivity and finally the H-score rating.

### 2.12 Single-cell data preprocessing and clustering annotation

High-throughput sequencing expression profile data (GSE136103) were downloaded from the Gene Expression Omnibus (GEO) database (http://www.ncbi.nlm.nih.gov/) (29). Twenty liver tissue samples were selected, including 9 MAFLD patients and 11 healthy individuals. Doublets in each sample were identified using DoubletFinder (30). Subsequently, doublets were filtered out, retaining cells meeting the following criteria: nFeature_RNA > 500 and < 3000; nCount_RNA < 10,000; percent_mito < 10. Cells from different samples were then merged using the merge function and analyzed using the Seurat package (31). Using the NormalizeData function, data were normalized for each library with the LogNormalize method and scale.factor of 10,000. The top 2,000 variable features were identified through the FindVariableFeatures function (32), and linear dimensionality reduction was performed using the RunPCA function with default parameters (npcs = 50). Batch effects were corrected using the RunHarmony function (33), followed by nonlinear dimensionality reduction using the RunUMAP function. The nearest neighbor graph was constructed using the FindNeighbors function (k.param = 20) with 30 principal components. Cell clustering was completed using the FindClusters function. Cluster marker genes were identified through the FindAllMarkers function with min.pct = 0.5 and logfc.threshold = 1. Single-cell samples were clustered and characterized using known lineage markers and manually annotated with reference to the human liver cell atlas on the CellMarker2 website (http://117.50.127.228/CellMarker/) (34). Differential expression analysis between cell clusters was performed using the FindMarkers function (Wilcoxon rank-sum test, p_val < 0.05 & abs(avg_log2FC) > 0.5).

### 2.13 Exploring cell clusters associated with MAFLD

To further explore intergroup heterogeneity across cell clusters, we analyzed proportional differences in cell counts between control and disease groups within distinct clusters, intergroup density variations, and distributional biases across conditions. Additionally, to test changes in cellular abundance at high resolution between groups/conditions, we employed the neighborhood abundance method in Milo (v2.2.0) (35) using the functions “buildGraph” and “makeNhoods” with parameters: “K=30”, “d=50”, “prop=0.2”, “refined=TRUE”, and “refinement_scheme=reduced_dim”, indicating sampling using dimensionality-reduced data, with remaining parameters selected according to Milo’s standard workflow recommendations. For pathway activity scoring of each cell cluster, we used the R package AUCell (v1.28.0): first, gene expression rankings per cell were computed via AUCell_buildRankings using the expression matrix with default parameters; then, custom-defined gene sets constructed from key genes identified in bulk-RNA analyses were applied to score individual cells; during this process, for each cell, the Area Under the Curve (AUC) value was calculated with AUCell_calcAUC based on gene expression rankings, where the AUC value represents the proportion of genes in the custom-defined set appearing among top-ranked genes per cell (36). Gene Set Variation Analysis implemented in the GSVA package (v2.0.0) was applied for gene set enrichment analysis using the “HALLMARK gene set” exported via GSEABase (v1.44.0). Intergroup differences in pathway activity scores per cell cluster between control and disease groups were computed using the LIMMA package (v3.62.2). Finally, based on intergroup heterogeneity patterns across cell clusters, we extracted Liver Sinusoidal Endothelial Cells (LSECs) and repeated the standard Seurat V5 workflow on these cells to identify and visualize endothelial subpopulations exhibiting significant distributional differences between groups.

### 2.14 Pseudo-temporal analysis

Unsupervised pseudotemporal analysis was performed using the “Monocle” package (v2.34.0) (37). First, a cell dataset containing the expression matrix, phenotypic data, and feature data was constructed with the newCellDataSet function (expressionFamily = negbinomial.size). Next, size factor dispersion and gene expression across cells were corrected via estimateSizeFactors and estimateDispersions functions. Subsequently, dimensionality reduction was conducted using the DDRTree method (parameter max_components = 2), and cell ordering and visualization were performed with plot_cell_trajectory (38), while filtering highly variable genes correlated with pseudotime; expression changes of these genes along pseudotime were analyzed using the differentialGeneTest function. Finally, the filtered genes were clustered into distinct groups based on expression patterns and visualized using plot_pseudotime_heatmap. To identify genes bifurcating cells into different branches while exploring specific roles of key genes from bulk RNA analyses in single-cell pseudotime trajectories, we conducted “Branched Expression Analysis Modeling” (BEAM) analysis (39), further enhancing the filtering threshold; genes from BEAM analysis were visualized via plot_genes_branched_heatmap. Subsequently, KEGG functional enrichment analysis was performed for genes within each cluster using clusterProfiler (v4.14.4) and org.Hs.eg.db (v3.20.0) packages.

### 2.15 Cell–cell communication

Initially, to further explore the LSEC clusters derived from prior research that may be closely associated with MAFLD progression, we processed and re-clustered LSECs using the standard Seurat V5 workflow. Then, by comparing these clusters through UMAP visualization of MAFLD and healthy control groups, the LSEC subpopulation exhibiting the most distinct cellular distribution was identified. Additionally, beyond LSECs, we identified cell clusters specifically distributing “bulk-RNA key genes” through scatter plots of gene distribution across cells; these cell clusters were simultaneously analyzed with LSECs for cell-cell communication analysis using the “CellChat” package (v2.1.2) (40). A random seed was set (seed=0528), and 8000 cells were randomly selected to create a CellChat object. Ligand-receptor interactions of “secretory signaling” were analyzed using “human” data from CellChatDB. The netVisual_circle function displayed the number and strength of inter-cell connections, while the netVisual_bubble function illustrated communications between different cell types. Subsequently, the netVisual_aggregate function demonstrated the communication network of a specific signaling pathway and calculated the contribution of various ligands. The mutual communication network between cells was visualized using the netVisual_individual function. Network centrality analysis was conducted using the netAnalysis_computeCentrality function (41) and represented as a heatmap.

### 2.16 Statistical analyses

Data analysis utilized SPSS 26.0 statistical software. Normally distributed measurement data were expressed as mean ± standard deviation and compared between two groups using independent samples t-test. Non-normally distributed measurement data were presented using the median and interquartile range, and comparisons between groups were conducted using the Mann-Whitney test. Statistical significance was set at P < 0.05.

## 3 Results

### 3.1 Screening of DEGs

**Figure 1:**
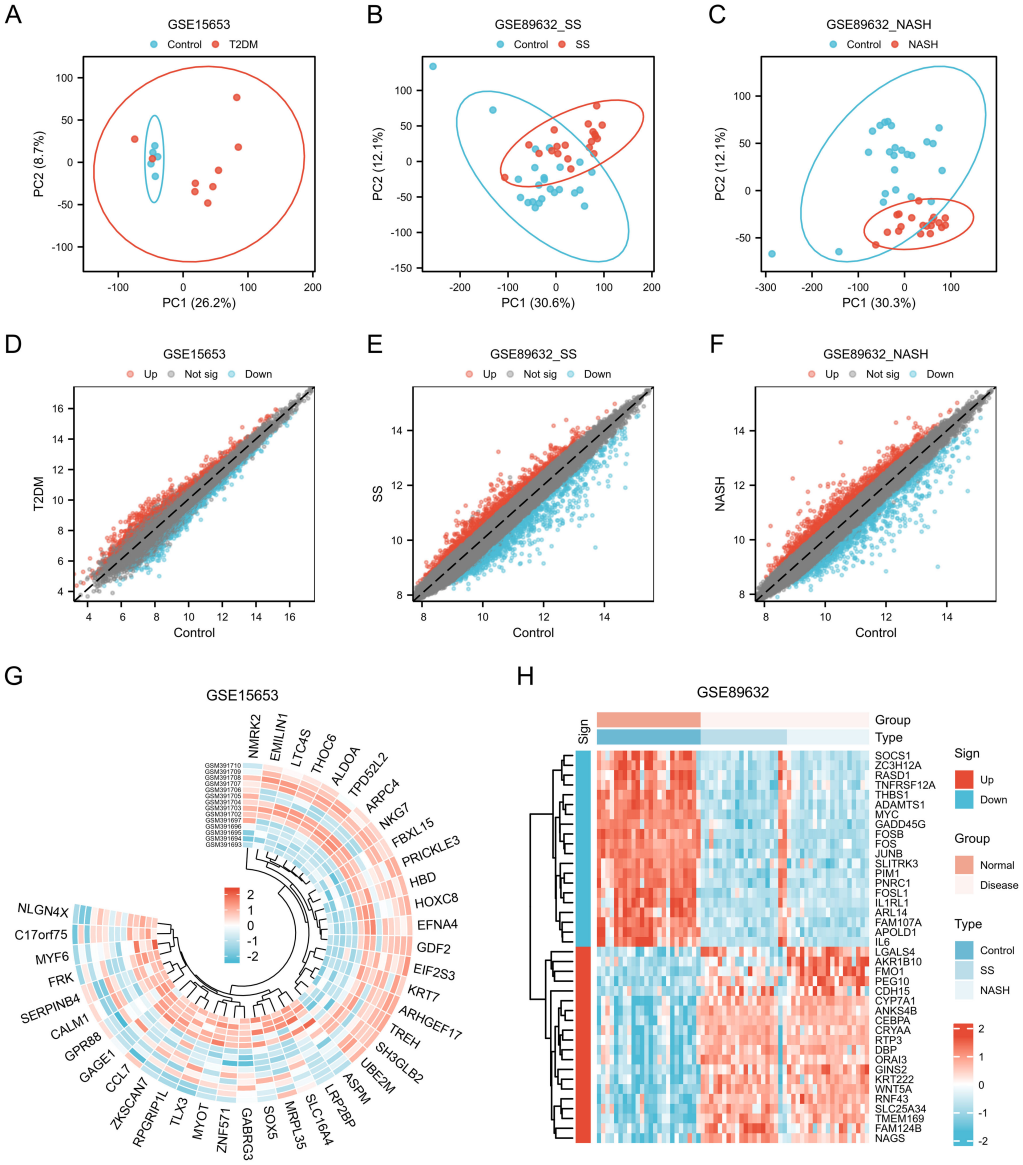
Genes of interest. **(A–C)** Principal component analysis of GSE15653, GSE89632_SS and GSE89632_NASH. **(D–F)** Volcano plots of differentially expressed mRNAs in GSE15653, GSE89632_SS, and GSE89632_NASH, |logFC| > 0.5, P < 0.05. **(G, H)** Expression heatmaps of the top40 DEGs in the GSE15653 and GSE89632 datasets.

The datasets GSE15653 and GSE89632 were downloaded from GEO data. The GSE89632 dataset was divided into GSE89632_SS group and GSE89632_NASH group according to SS and NASH samples. PCA principal component analysis was performed separately, and in the GSE15653 dataset, PC1 was 26.2% and PC2 was 8.7% (**Figure 1A**); in the GSE89632_SS dataset, PC1 was 30.6% and PC2 was 12.1% (**Figure 1B**); in the GSE89632_NASH dataset, PC1 was 30.3% and PC2 was 12.1% (**Figure 1C**). The volcano plot showed that 1686 differentially expressed genes were identified in the GSE15653 dataset with |log2(FC)|>0.5, p-value<0.05 as the screening threshold, of which 864 were up-regulated and 822 were down-regulated (**Figure 1D**). In the GSE89632_SS dataset, 2219 differentially expressed genes were identified, of which 1003 genes were up-regulated in expression and 1216 genes were down-regulated in expression, as in (**Figure 1E**). In the GSE89632_NASH dataset, 2248 differentially expressed genes were identified, of which 1062 genes were up-regulated in expression and 1186 genes were down-regulated in expression, as in (**Figure 1F**). The ring value heatmap and complex value heatmap showed the genes ranked top40 in differential folds in GSE15653 (**Figure 1G**), GSE89632_SS (**Figure 1H**) and GSE89632_NASH (**Figure 1H**), respectively.

### 3.2 WGCNA analysis

**Figure 2:**
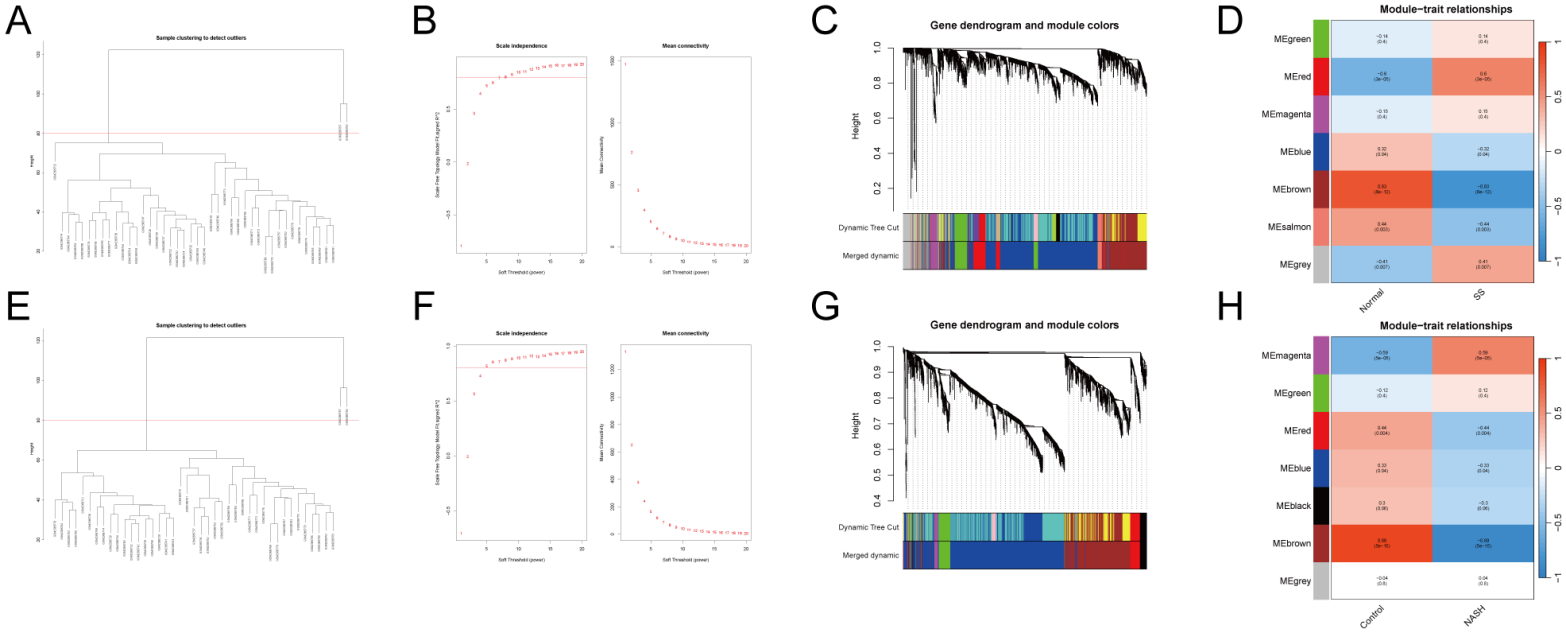
WGCNA.**(A–D)** GSE89632_SS.**(E–H)** GSE89632_NASH.**(A, E)** Sample dendrogram with outlier samples above the red line. **(B, F)** Scale-free fit indices and average connectivity for different soft thresholds. **(C, G)** Clustering dendrogram after fusing similar modules. **(D, H)** Module-trait association plot.

Weighted gene co-expression network analysis (WGCNA) was performed on GSE89632*SS*, *GSE89632*NASH. Hierarchical clustering was performed based on similarity or correlation between samples and visualized as a hierarchical dendrogram (**Figures 2A, E**). The distance between samples indicated the degree of similarity or correlation between samples, and outlier samples were removed by pruning operation, and the outlier samples rejected were GSM2385767 and GSM2385782, and the most representative subset of samples was selected, and the clustering differences between different samples in the Normal group and the MAFLD group were visualized in a joint heat map (**Figures 2C, G**). The top 25% of genes with the largest fluctuations were selected for WGCNA analysis based on standard deviation ranking. The scale-free fit indices and average connectivity of various soft-threshold powers were evaluated on the basis of scale-free R2. Among them, GSE89632_SS selected soft threshold powers with β = 11 and scale-free R2 = 0.8 (**Figure 2B**), and *GSE89632*NASH selected soft threshold powers with β = 5 and scale-free R2 = 0.8 (**Figure 2F**). Gene clustering tree with module identification demonstrates the results of hierarchical clustering of genes in the two datasets and categorizes genes into different modules, further merging similar modules to reduce redundancy (**Supplementary Figures 2A, D**). The hierarchical clustering results between different modules were visualized using module clustering dendrogram (**Supplementary Figures 2B, E**), while the correlations between different modules were visualized with modal correlation heatmap (**Supplementary Figures 2C, F**). Finally, GSE89632*SS identified 7 modules* (**Figure 2D**), GSE89632_NASH identified 7 modules (**Figure 2H**). We performed KEGG enrichment analysis on genes within the "brown" modules of GSE89632_SS and GSE89632_NASH. The results demonstrated that in the SS group, pathways including TNF-JNK signaling pathway,IL-1/IL-1R-JNK signaling cascade, and TLR2/4-MAPK signaling axis collectively indicate the role of pro-inflammatory signaling in driving steatosis progression toward fibrosis. Concurrently, pathways such as Environmental factor-induced PI3K signaling pathway and Environmental factor-mediated RAS-ERK signaling cascade (activated by heavy metals) suggest synergistic interactions between environmental carcinogens and metabolic dysregulation. In the NASH group, the recurrent enrichment of TNF-JNK signaling pathway and Environmental factor-mediated RAS-ERK signaling cascade further validates conserved mechanisms underlying inflammation-carcinogenesis transition. The observed negative correlation may reflect compensatory dysregulation of protective mechanisms during disease progression, where downregulation of module genes attenuates their inhibitory effects on pro-inflammatory/carcinogenic pathways, thereby accelerating pathological advancement (**Supplementary Files 1**, **2**). Based on this observation, we selected the "brown" modules from both SS and NASH WGCNA cohorts.

### 3.3 Protein-protein interaction network

**Figure 3:**
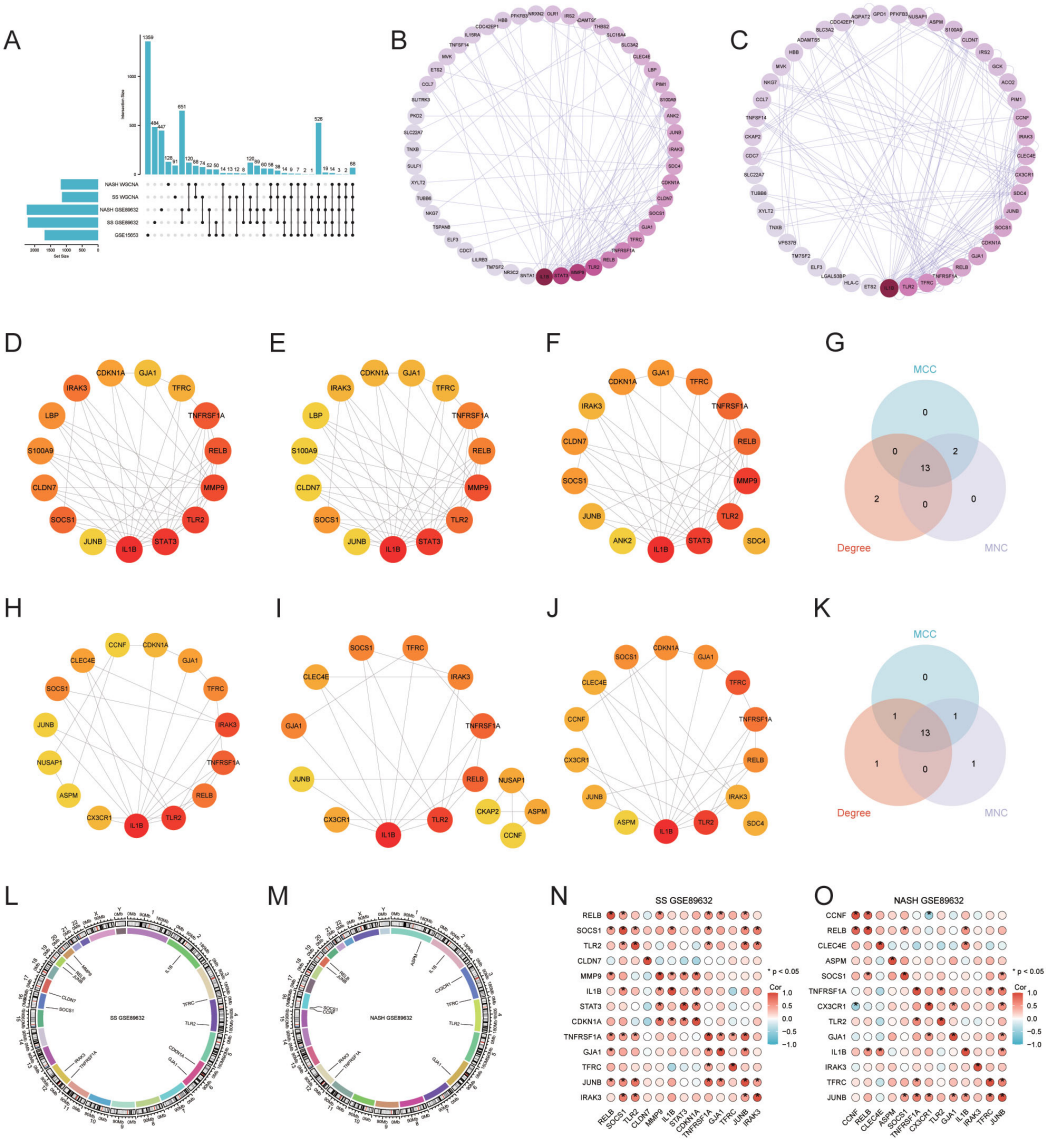
Protein-Protein Interaction (PPI) Network and 13 Hub Genes. **(A)** UpSet Plot. **(B, C)** PPI networks for GSE89632_SS and GSE89632_NASH. **(D–F)** GSE89632_SS: Top 15 genes identified by the MCC, MNC, and Degree algorithms. **(H–J)** GSE89632_NASH: Top 15 genes identified by the MCC, MNC, and Degree algorithms. **(G)** GSE89632_SS: Venn diagrams illustrating the intersecting genes across the three algorithms. **(K)** GSE89632_NASH: Venn diagram depicting the intersection of genes identified by the three algorithms. **(L)** GSE89632_SS: Chromosomal localization of 13 hub genes. **(M)** GSE89632_NASH: Chromosomal localization of 13 hub genes. **(N)** GSE89632_SS: Correlation heatmap of 13 hub genes. **(O)** GSE89632_NASH: Correlation heatmap of 13 hub genes.

UpSet plots visualize the results of intersections of DEGs of GSE15653 taken with GSE89632_SS, GSE89632_NASH, SS WGCNA, NASH WGCNA, respectively. (**Figure 3A**). In order to better understand the interactions between genes, we used the STRING database and constructed the PPI network (**Figures 3B, C**), while the CytoHubba plugin was used to identify the top 15 hub genes in the GSE89632_SS group and the GSE89632_NASH group, respectively, based on the MCC, MNC, and Degree algorithms (**Figures 3D–F**, **3H–J**), and then these three algorithms took the intersection to obtain 13 hub genes each (**Figures 3G**, **K**). Next, we investigated the chromosomal localization (**Figures 3L**, **M**) and interrelationships (**Figures 3N**, **O**) of the 13 hub genes.

### 3.4 Shared screen of multiple machine learning algorithms

**Figure 4:**
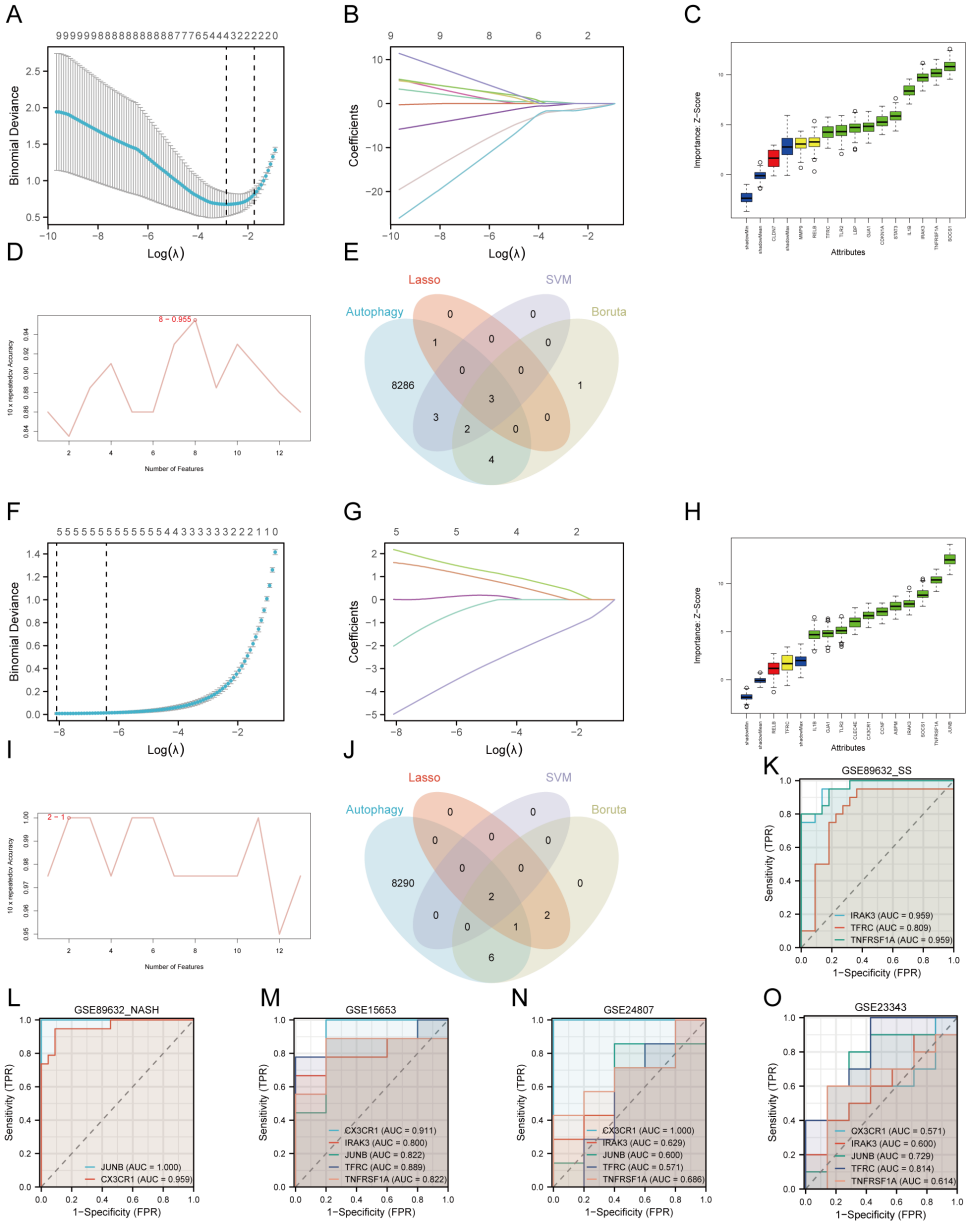
Machine learning screening biomarker. **(A–E)** Dataset GSE89632_SS. **(F–J)** Dataset GSE89632_NASH. **(A, F)** Cross validation of parameter selection in Lasso regression. **(B, G)** Lasso regression for 13 hub genes. **(C, H)** Boruta algorithm feature gene screening. **(D, I)** SVM-RFE algorithm feature gene screening. **(E, J)** Wayne plots. **(K)** ROC curves for IRAK3, TFRC, TNFRSF1A in GSE89632_SS. **(L)** ROC curves for CX3CR1, JUNB in GSE89632_NASH. **(M)** ROC curves for IRAK3, TFRC, TNFRSF1A, CX3CR1, JUNB in GSE15653. **(N)** ROC curves for IRAK3, TFRC, TNFRSF1A, CX3CR1, JUNB in GSE24807. **(O)** ROC curves for IRAK3, TFRC, TNFRSF1A, CX3CR1, JUNB in GSE23343. AUC, area under the curve; TPR, true positive rate; FPR, false positive rate.

We employed Lasso, Boruta, and RFE-SVM algorithms to identify hub genes associated with T2DM and MAFLD in the GSE89632_SS and GSE89632_NASH datasets. In GSE89632_SS, Lasso analysis revealed four significant variables: CLDN7, IRAK3, TFRC, and TNFRSF1A (**Figures 4A, B**). The Boruta algorithm identified ten significant variables, including CDKN1A, GJA1, IL1B, IRAK3, LBP, SOCS1, STAT3, TFRC, TLR2, and TNFRSF1A (**Figure 4C**). RFE_SVM highlighted eight features: IRAK3, MMP9, TNFRSF1A, TLR2, JUNB, TFRC, RELB, and CDKN1A (**Figure 4D**). In GSE89632_NASH, Lasso analysis identified five significant variables: ASPM, CCNF, CLEC4E, CX3CR1, and JUNB (**Figures 4F, G**). Furthermore, the Boruta algorithm filtered eleven significant variables, specifically ASPM, CCNF, CLEC4E, CX3CR1, GJA1, IL1B, IRAK3, JUNB, SOCS1, TLR2, and TNFRSF1A (**Figure 4H**). Two features were identified through RFE_SVM: JUNB and CX3CR1 (**Figure 4I**). Subsequently, we intersected the machine learning results from both datasets with autophagy-related genes to derive diagnostic biomarkers associated with autophagy in type 2 diabetes mellitus combined with NAFLD (**Figures 4E, J**).

### 3.5 Performance of five diagnostic biomarkers

We assessed the diagnostic performance of IRAK3, TFRC, TNFRSF1A, CX3CR1, and JUNB using the datasets GSE89632_SS, GSE89632_NASH, and GSE15653 for training, followed by plotting ROC curves. The results indicated that in GSE89632_SS, the AUC for IRAK3, TFRC, and TNFRSF1A exceeded 0.8 (**Figure 4K**). In GSE89632_NASH, the AUC for CX3CR1 and JUNB also surpassed 0.8 (**Figure 4L**), reflecting their high predictive accuracy. In GSE15653, the AUCs for all five genes were above 0.8 (**Figure 4M**). Subsequently, we conducted external validation using independent datasets GSE24807 and GSE23343. The results showed that in GSE24807, the AUC values of IRAK3, TNFRSF1A, CX3CR1, and JUNB were all greater than 0.6, while in GSE23343, the AUC values of IRAK3, TNFRSF1A, CX3CR1, and JUNB were all greater than 0.5 (**Figures 4N, O**). These results indicate that in the two independent external validation sets, these indicators also possess certain diagnostic value, which is consistent with our previous predictions. Ultimately, we identified IRAK3, TNFRSF1A, CX3CR1, and JUNB as diagnostic biomarkers for T2DM complicated with MAFLD associated with autophagy and endoplasmic reticulum stress.

### 3.6 Enrichment analysis of GO and KEGG

**Figure 5:**
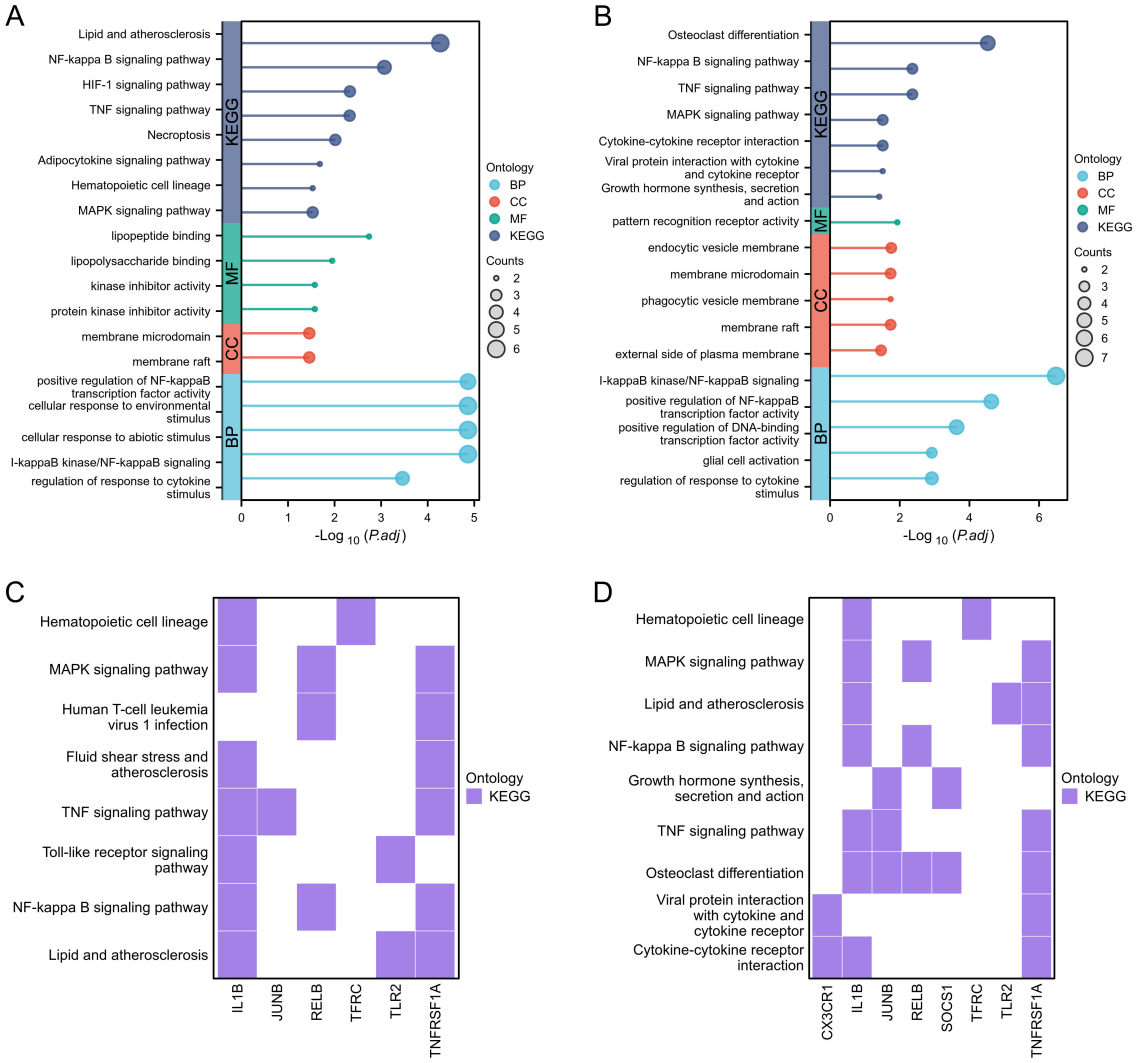
Functional enrichment analysis. **(A, B)** Lollipop diagrams showing GO and KEGG enrichment analysis of GSE89632_SS group and GSE89632_NASH group, respectively. **(C, D)** Heatmaps showing the pathway enrichment of some hub genes in GSE89632_SS and GSE89632_NASH groups, respectively.

During the GO and KEGG enrichment analysis, TNFRSF1A and JUNB were predominantly enriched in the TNF signaling pathway, while TNFRSF1A and TFRC showed significant enrichment in the HIF-1 signaling pathway. Furthermore, TNFRSF1A demonstrated substantial enrichment in the NF-kappa B signaling pathway, MAPK signaling pathway, Fluid shear stress and atherosclerosis, and Insulin resistance (**Figure 5A**, **Figure 5C**). CX3CR1 exhibited notable enrichment in the Cytokine-cytokine receptor interaction pathway, and TFRC displayed significant enrichment in the Hematopoietic cell lineage (**Figures 5B, D**).

### 3.7 Enrichment analysis of GSEA

In Enrichment analysis of GSEA, IRAK3、TNFRSF1A 和 JUNB commonly enriched in REACTOME_SIGNALING_BY_INTERLEUKINS(**Supplementary Figure 3A**, **Supplementary Figure 3E**, **Supplementary Figure 3M**).IRAK3 mainly enriched in BIOCARTA_IL1R_PATHWAY(**Supplementary Figure 3B**);REACTOME_TOLL_LIKE_RECEPTOR_TLR1_TLR2_CASCADE(**Supplementary Figure 3C**);REACTOME_TOLL_LIKE_RECEPTOR_CASCADES (**Supplementary Figure 3D**).TNFRSF1A mainly enriched in KEGG_CYTOKINE_CYTOKINE_RECEPTOR_INTERACTION (**Supplementary Figure 3F**);REACTOME_INTERLEUKIN_10_SIGNALING (**Supplementary Figure 3G**);KEGG_MAPK_SIGNALING_PATHWAY(**Supplementary Figure 3H**).TFRC mainly enriched in PID_HIF1_TFPATHWAY(**Supplementary Figure 3I**);KEGG_HEMATOPOIETIC_CELL_LINEAGE (**Supplementary Figure 3J**). JUNB mainly enriched in REACTOME_INTERLEUKIN_4_AND_INTERLEUKIN_13_SIGNALING(**Supplementary Figure 3K**);WP_TGFBETA_SIGNALING_PATHWAY(**Supplementary Figure 3L**); PID_IL6_7_PATHWAY(**Supplementary Figure 3N**);REACTOME_SIGNALING_BY_TGF_BETA_RECEPTOR_COMPLEX (**Supplementary Figure 3O** **).**

### 3.8 Infiltration and functional exploration of immune cells

**Figure 6:**
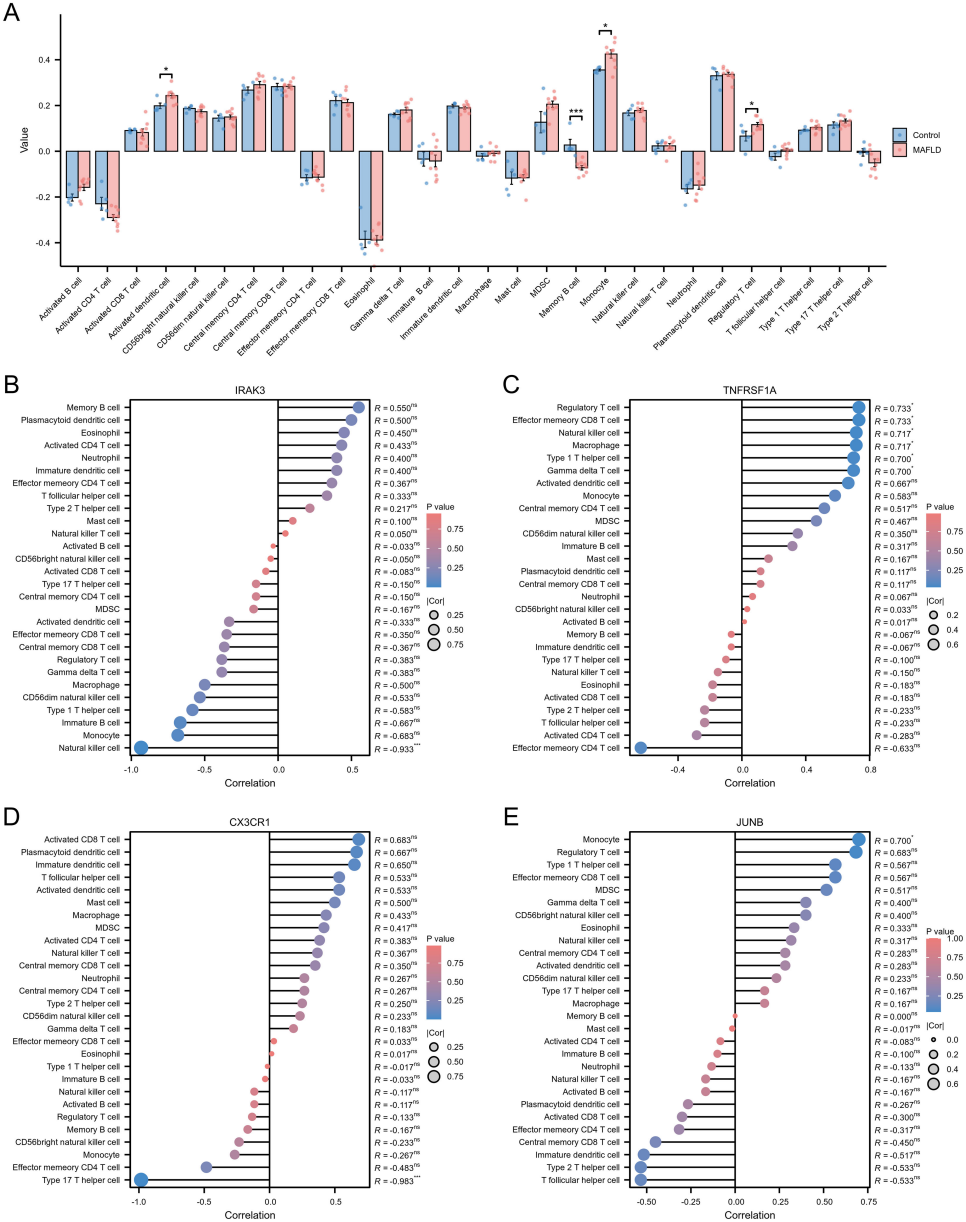
Immune cell infiltration assessment. **(A)** Subgroup comparison plot demonstrating the difference in immune cell infiltration between the two groups as calculated by the ssGSEA algorithm. **(B–E)** Lollipop plots showing the correlation between IRAK3 **(B)**, TNFRSF1A **(C)**, CX3CR1 **(D)**, JUNB **(E)** and immune cells. Note: Significance levels are denoted as follows: ns p ≥0.05; * p < 0.05; ** p < 0.01; *** p < 0.001.

To assess immune cell infiltration and functional differences between the T2DM and control groups, we utilized the ssGSEA method to evaluate various immune cell subpopulations. The group comparison plot revealed increased levels of Activated dendritic cells, Monocytes, and Regulatory T cells in the T2DM group, with decreased levels of Memory B cells (**Figure 6A**). The correlation coefficient’s absolute value indicates the strength of correlation: 0.3-0.5 denotes weak correlation, 0.5-0.8 signifies moderate correlation, and 0.8–1 represents strong correlation; P < 0.05 indicates statistical significance. In the T2DM group, IRAK3 exhibited a negative correlation with Natural killer cell infiltration level (R=-0.933) (**Figure 6B**, **Supplementary Figure 4A**). The correlations between TNFRSF1A and Effector memory CD8T cells, Gamma delta T cells, Macrophages, Natural killer cells, Type 1 T helper cells, and Regulatory T cells were positive (R=0.733, 0.700, 0.717, 0.717, 0.700, 0.733) (**Figure 6C**, **Supplementary Figures 4B–G**). CX3CR1 showed a negative correlation with Type 17 T helper cell infiltration (R=-0.983) (**Figure 6D**, **Supplementary Figure 4H**). JUNB displayed a positive correlation with Monocyte infiltration (R=0.700) (**Figure 6E**, **Supplementary Figure 4I**). Additionally, correlations existed between different types of immune cells (**Supplementary Figure 4J**).

### 3.9 Real-time fluorescence quantitative PCR results

**Figure 7:**
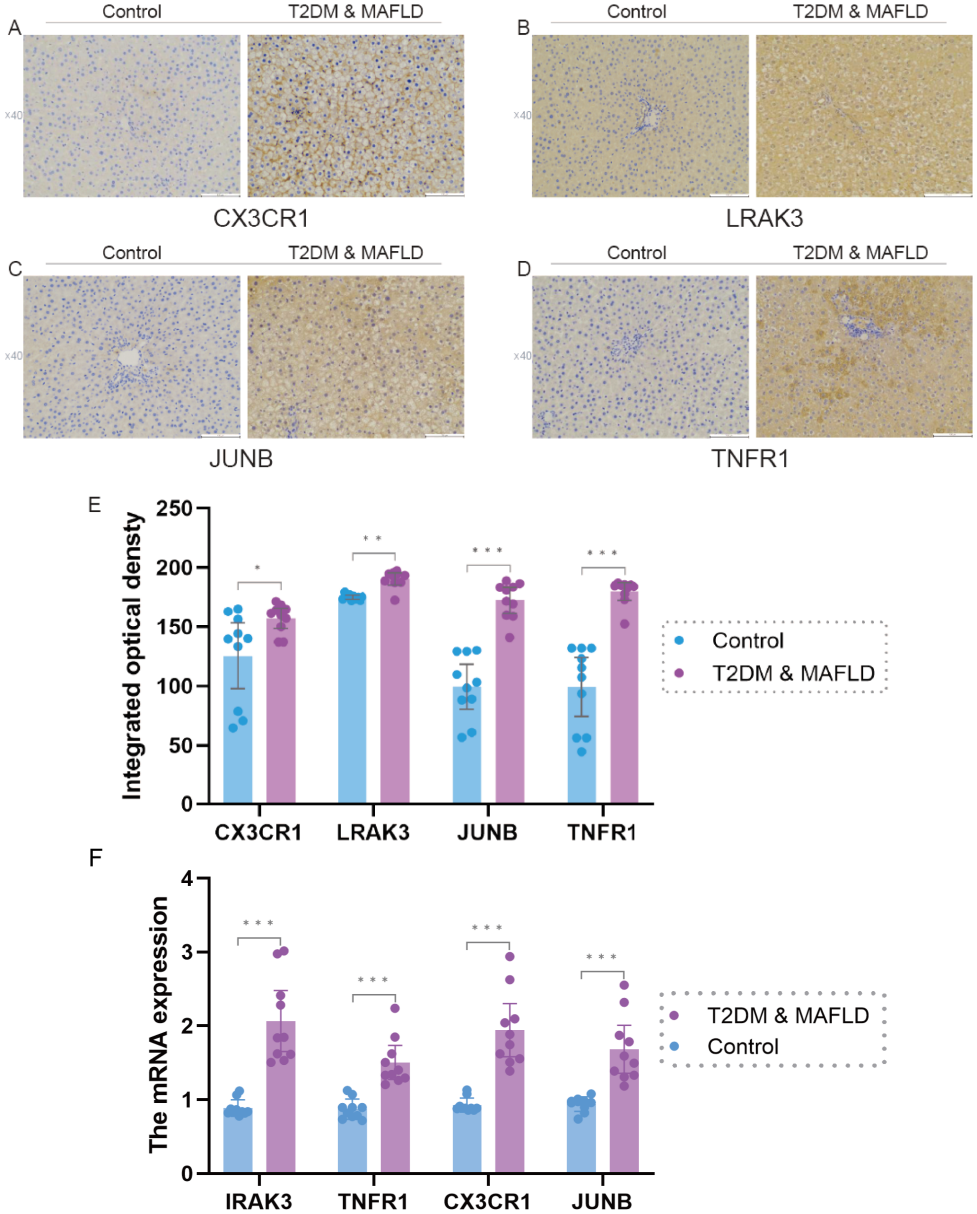
**(F)**. IRAK3, TNFR1,CX3CR1, JUNB mRNA expression in liver tissues of rats in T2DM and MAFLD group and control group. **(A–E)**, Expression levels of antigen-antibody complexes in the liver tissues of rats in the T2DM and MAFLD groups and the Control group. Note: Significance levels are denoted as follows: ns p ≥0.05; * p < 0.05; ** p < 0.01; *** p < 0.001.

The results of real-time fluorescence quantitative PCR analysis showed that there were significant differences in the expression levels of IRAK3, TNFR1, CX3CR1 and JUNB between the two groups (P < 0.05); among them, the mRNA expression levels of IRAK3, TNFR1, CX3CR1 and JUNB in the liver tissues of rats in the T2DM combined with MAFLD group were significantly higher than that in the control group (P > 0.05).), as shown in **Figure 7F**.

### 3.10 Immunohistochemical results

Immunohistochemical picture analysis showed that the expression levels of the four factors, CX3CR1, IRAK3, JUNB, and TNFR1, were significantly different between the T2DM & MAFLD groups and the Control group (P < 0.05); and the T2DM combined with MAFLD group were significantly higher than the Control group. For details, see **Supplementary Table S3** and **Figures 7A–E**.

### 3.11 Single-cell data preprocessing and clustering annotation

**Figure 8:**
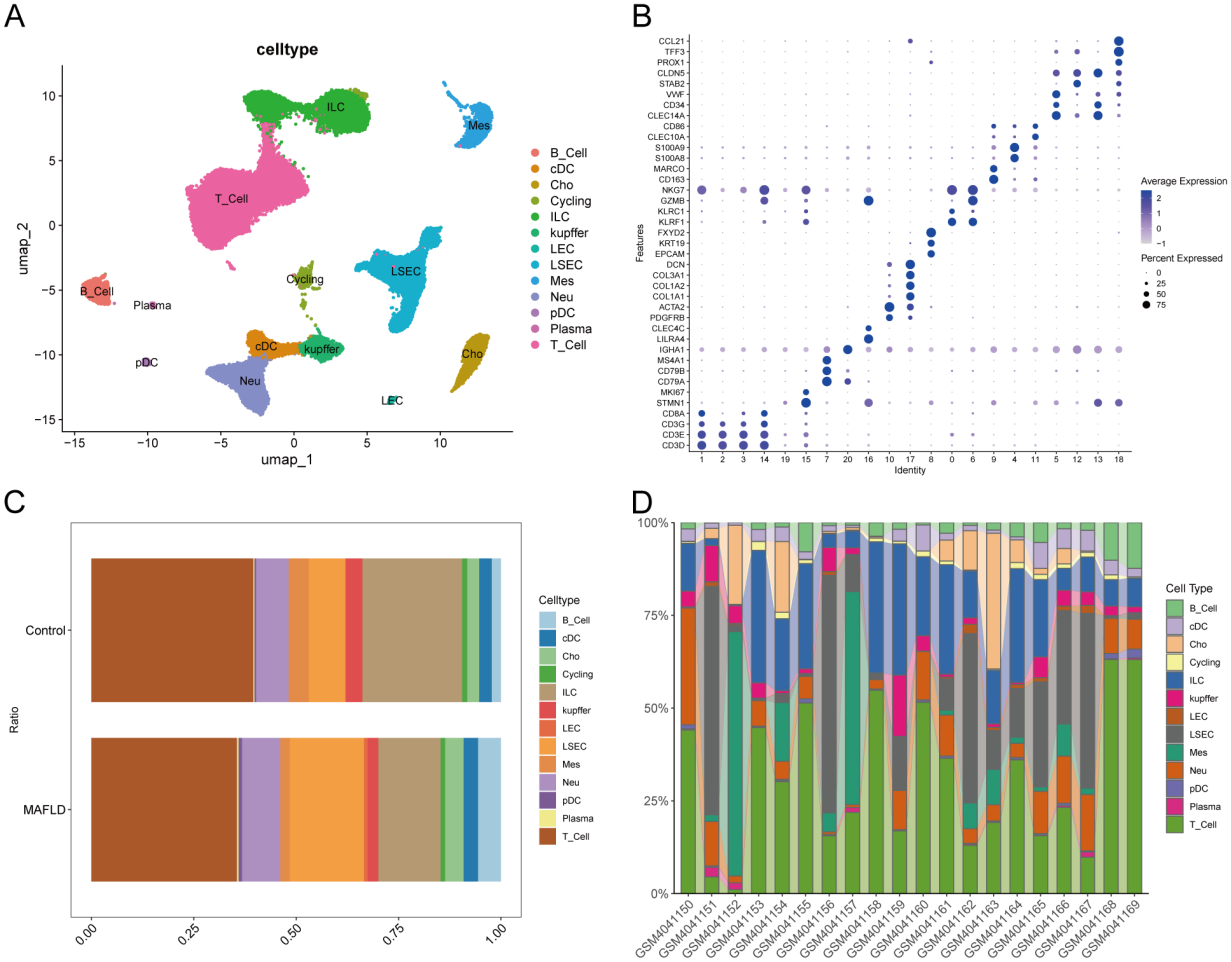
Cell types annotation. **(A)** UMAP plot showing the clustering results of cells at 0.6 resolution. **(B)** Bubble diagram showing Marker genes used to identify major cell types. **(C)** Percentage of different cell types between Control and MAFLD groups. **(D)** Percentage of different cell types among different samples between Control and MAFLD groups.

A comprehensive scRNA-seq analysis was conducted using publicly available data from healthy and MAFLD liver samples. The clustering results, illustrated through dendrograms(**Supplementary Figure 5A**) and marker genes bubble plots (**Figure 8B**), culminated in the identification of 13 major cell clusters at a resolution of 0.6. Distinct cell types were pinpointed, including Cycling(cycling cells) (STMN1,MKI67), B_cell (CD79A,CD79B,MS4A1), Plasma(Plasma cell) (CD79A,IGHA1), pDC (plasmacytoid dendritic cell) (LILRA4,CLEC4C), Mes(mesenchymal cell) (PDGFRB,ACTA2,COL1A1,COL1A2,COL3A1,DCN), Cho (Cholangiocyte) (EPCAM,KRT19,FXYD2), T_cell (CD3D,CD3E,CD3G,CD8A), ILC (innate lymphoid cell) (KLRF1,KLRC1,GZMB,NKG7), Kupffer (CD163,MARCO), Neu(Neutrophils) (S100A8,S100A9), cDC(Conventional Dendritic Cell) (CLEC10A,CD86), LSEC(Liver Sinusoidal Endothelial Cel) (CLEC14A,CD34,VWF,STAB2,CLDN5) and LEC(Lymphatic Endothelial Cells) (PROX1,TFF3,CCL21). The findings were further visualized using UMAP (**Figure 8A**).

### 3.12 Exploring cell clusters associated with MAFLD

**Figure 9:**
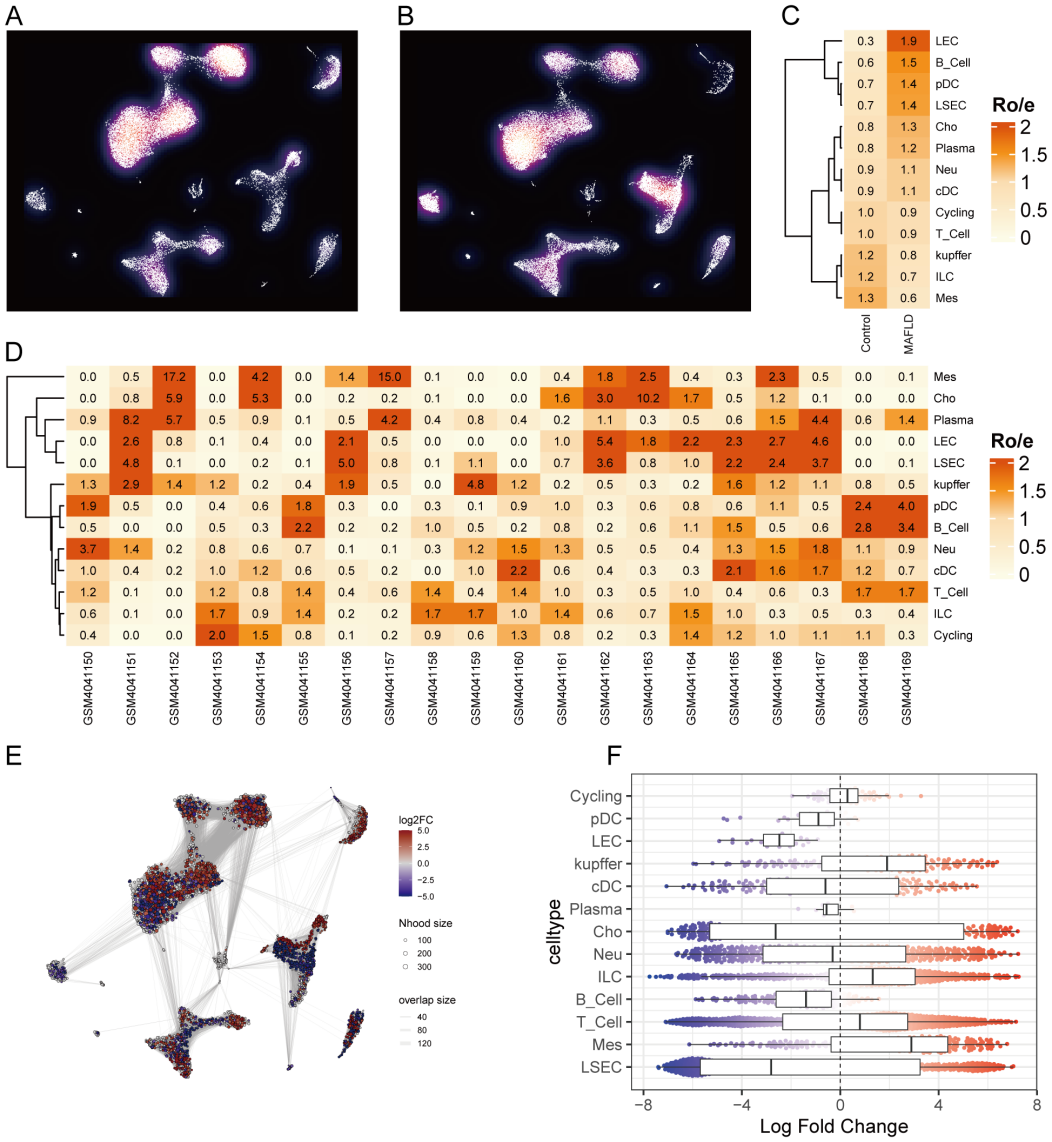
Cell clusters associated with MAFLD. **(A, B)** Density plots. **(C)** Distributional preference of different cells between groups evaluated by the Ro/e index. **(D)** Distributional preference of different cells across samples assessed by the Ro/e index. **(E)** Neighborhood graph of cells generated using Milo differential abundance testing. Nodes represent neighborhoods across different cell cluster populations. Color indicates the fold-change between MAFLD patients and healthy donors. Increased neighborhoods are shown in red, decreased neighborhoods in blue. **(F)** Beeswarm and box plots display the distribution of fold-change across neighborhoods in different cell type populations. Color coding follows the scheme in **(E)**.

**Figure 10:**
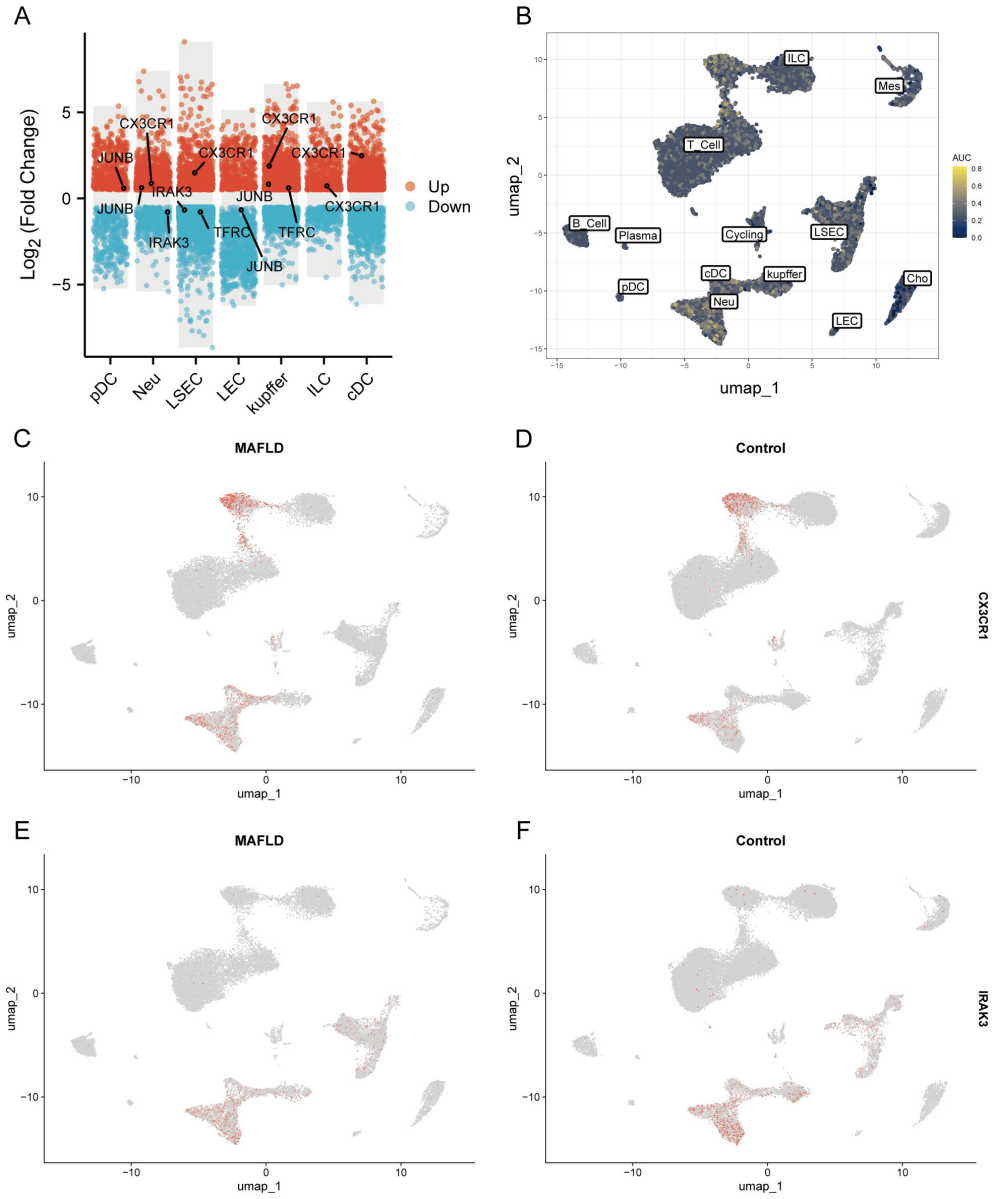
Differential expression analysis of different cell types and AUCell analysis. **(A)** Multi-subgroup volcano plots demonstrating differentially expressed genes in different cells between MAFLD and Control groups. **(B)** AUCell scores showing activity levels of the custom gene set across distinct cell populations. **(C, D)** CX3CR1 distribution plots in disease and control groups. **(E, F)** IRAK3 distribution plots in disease and control groups.

**Figure 11:**
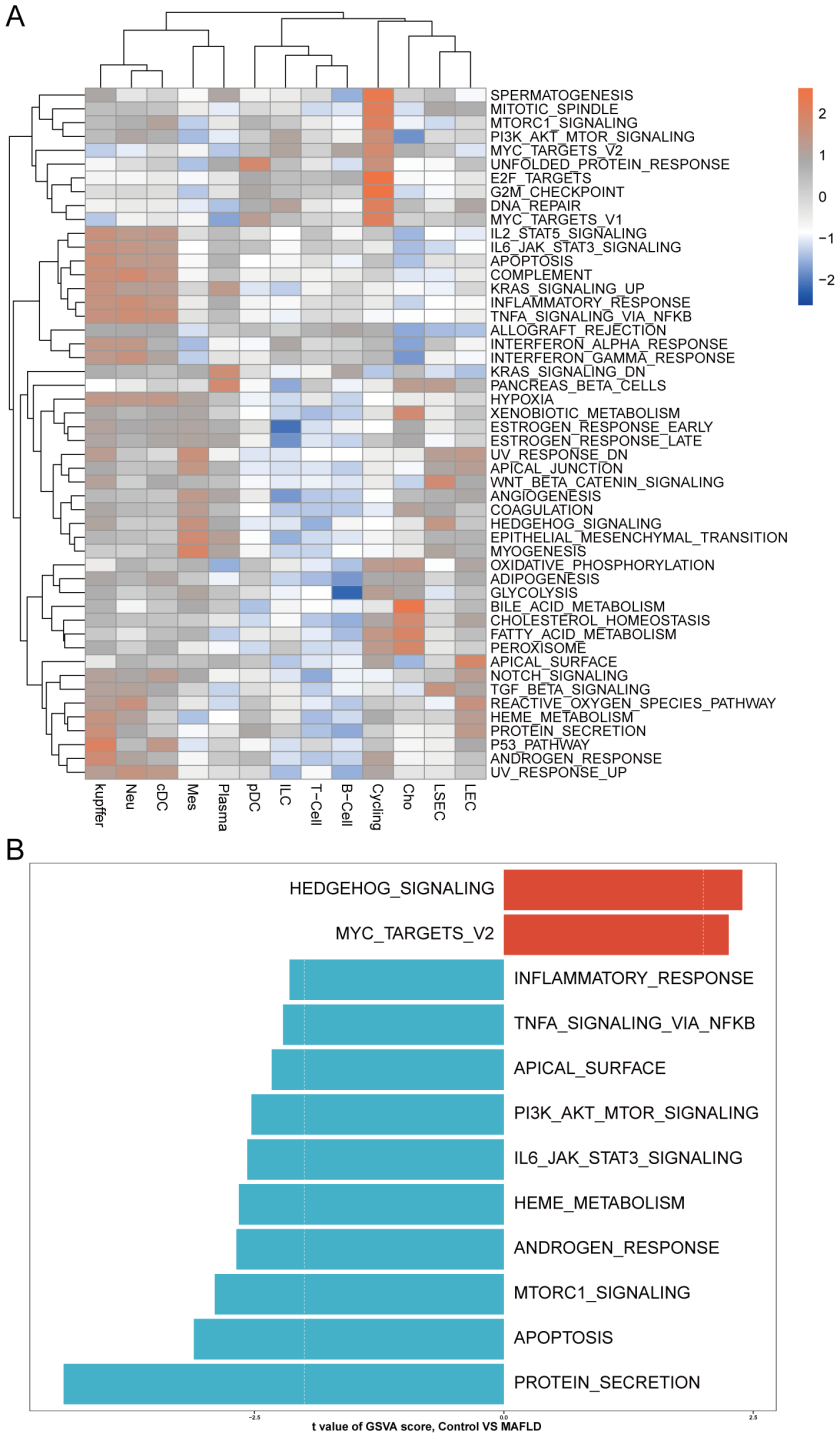
GSVA. **(A)** Heatmap displays enriched pathways from HALLMARK gene sets in MAFLD group cell clusters through GSVA analysis, with color representing z-score transformed mean GSVA scores. **(B)** Pathway activity differences between MAFLD and normal samples in LSECs assessed via GSVA.

We analyzed the proportional differences in cell counts between control and disease groups across distinct cell clusters, with LSEC showing a significantly higher proportion in the MAFLD group (**Figure 8C**), along with proportional variations of cell clusters among different samples (**Figure 8D**). We performed density analysis of inter-group cell counts at a two-dimensional level, where relative brightness indicates higher density of the corresponding cell cluster in its respective group (**Figures 9A, B**). Subsequently, we evaluated the distributional preference between MAFLD and Control groups using the Ro/e index (**Figure 9C**), as well as distributional preferences across individual samples (**Figure 9D**), revealing LSEC exhibiting stronger enrichment tendencies in the disease group. Further, in the Milo differential abundance testing, we identified significant disease-state-driven changes occurring specifically in LSEC, with a negative Log Fold Change in relative abundance (**Figures 9E, F**). Despite LSEC’s preferential enrichment in the MAFLD group (**Figure 9C**), the actual abundance of LSEC cells was significantly reduced (**Figure 9F**), suggesting potential widespread LSEC activation or proliferation in MAFLD state alongside intrinsic heterogeneity within LSEC populations, where certain subpopulations may undergo suppression or reduction. Subsequently, we explored cellular heterogeneity through differential gene expression and enrichment scoring perspectives to elucidate connections between key genes and various cell clusters. Using thresholds of |avg_log2FC|>1 and p_val<0.05, the FindMarkers function identified differentially expressed genes suitable for a multi-group volcano plot (**Figure 10A**). The potential marker genes for diabetes-combined MAFLD, derived from bulk RNA analysis, displayed significant differences during single-cell differential analysis. Specifically, JUNB was upregulated in pDC, Neu, and kupffer, but downregulated in LEC; CX3CR1 was upregulated in Neu, LSEC, kupffer, ILC, and cDC; IRAK3 exhibited downregulation in Neu and LSEC. CX3CR1 (**Figures 10C, D**) and IRAK3 (**Figures 10E, F**) also showed distinct distributional variations across cell types, predominantly localized within five cell populations: LSEC, ILC, Neu, cDC, and kupffer. Additionally, using key genes including JUNB, CX3CR1, IRAK3, and TNFRSF1A to construct a custom gene set, we scored activity levels across cell clusters on this custom dataset. Results revealed relatively higher activity of the key gene-defined custom set within these five cell types: Neu, LSEC, kupffer, ILC, and cDC (**Figure 10B**). Finally, we focused our investigation on LSEC and the other four cell types (ILC, Neu, cDC, kupffer) for subsequent research. Through gene set variation analysis in LSEC, we enriched pathways including “TGF BETA SIGNALING”, “WNT BETA CATENIN SIGNALING”, and “HEDGEHOG SIGNALING” (**Figure 11A**). The activity of these pathways indicates that in MAFLD, LSECs likely reside in a highly dynamic microenvironment involved in regulating cell proliferation, differentiation, immunity, and cellular interactions. When comparing enrichment differences in LSEC between disease and MAFLD groups, we observed significantly higher enrichment of “HEDGEHOG SIGNALING” in the MAFLD group. Additionally, pathways including “MYC TARGETS V2”, “PI3K-AKT-MTOR SIGNALING”, “IL6-JAK-STAT3 SIGNALING”, “APOPTOSIS” and “PROTEIN SECRETION”—significantly associated with biological processes like cell proliferation, survival, metabolic regulation, immune response, inflammatory response, and programmed cell death—exhibited substantial inter-group enrichment differences (**Figure 11B**). These results demonstrate that LSECs likely play crucial roles in MAFLD pathogenesis, warranting further investigation into their specific mechanisms during MAFLD development.

### 3.13 Pseudo-temporal trajectory analysis of LSEC during MAFLD and BEAM analysis

**Figure 12:**
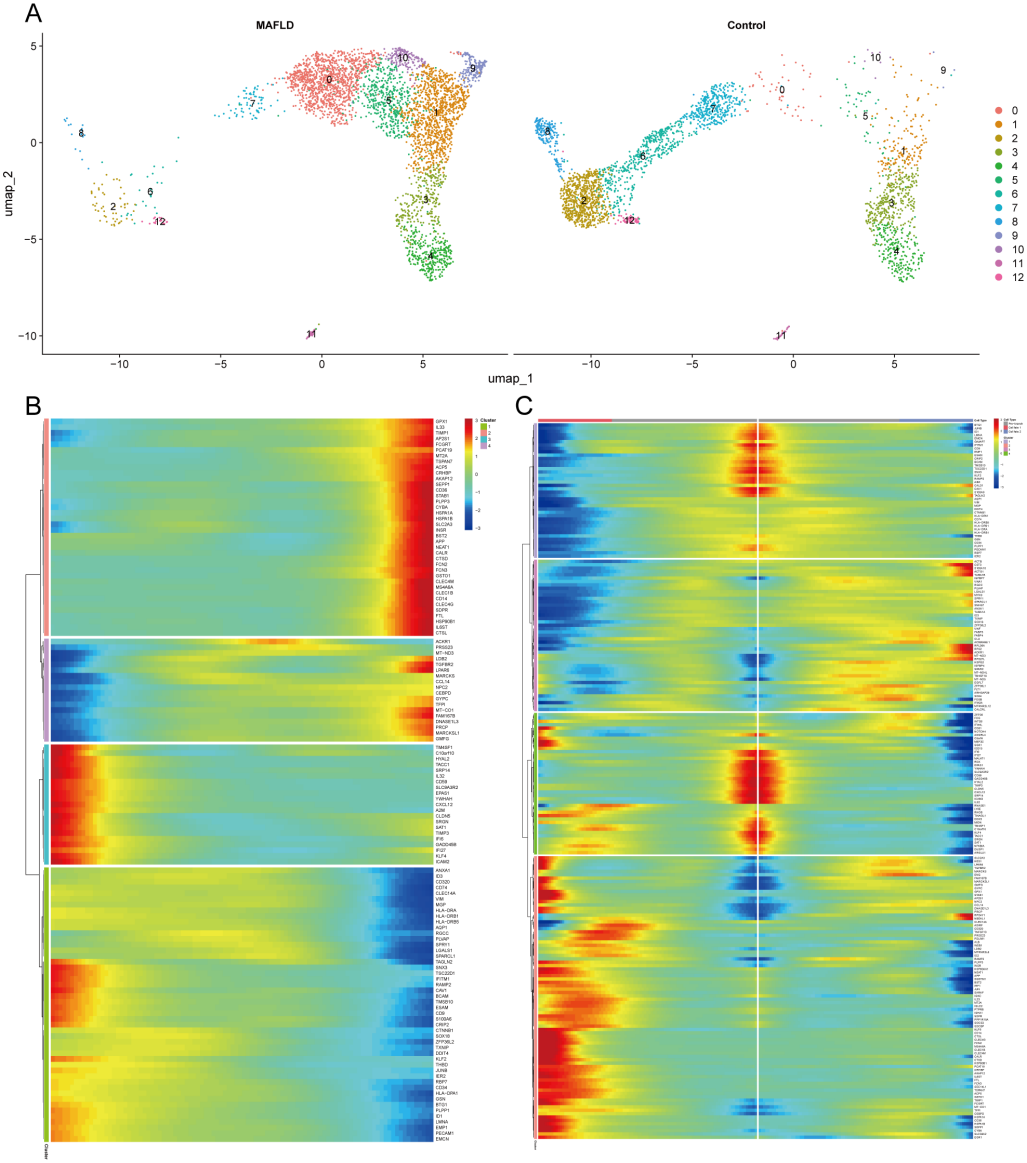
Clustering, enrichment analysis, and branching analysis of highly variable genes in the Pseudo-temporal process. **(A)** UMAP visualization of ECs re-clustering results in MAFLD group and Control group. **(B)** Four expression patterns of highly variable genes. **(C)** Four expression patterns of potential regulatory genes at branches.

Re-clustering of LSEC through secondary dimensionality reduction yielded 13 subpopulations, among which clusters 2, 6, 7, 8, and 12 showed significant reduction compared to the control group. This aligns with prior Milo analysis conclusions, indicating intrinsic heterogeneity within LSEC populations where specific subpopulations may be suppressed or diminished in the disease state. Conversely, clusters 0, 1, 5, 9, and 10 exhibited marked expansion. We defined the expanded clusters 0, 1, 5, 9, 10 as “LSEC_inc” and the reduced clusters 2, 6, 7, 8, 12 as “LSEC_red” (**Figure 12A**). We conducted a pseudo-temporal trajectory analysis on LSEC, dividing the entire trajectory into five stages (**Supplementary Figure 5B**), showing the distribution of different LSEC subgroups in the trajectory (**Supplementary Figure 5C**) and the direction of differentiation and development (**Supplementary Figure 5D**). The cell density plot along the timeline further illustrates the distribution and dynamic changes of LSEC during the pseudo-temporal process (**Supplementary Figure 5E**). There is a significant difference in the temporal trajectory distribution of LSEC between the Control group and the MAFLD group (**Supplementary Figure 5F**). Subsequently, we analyzed the expression patterns of highly variable genes in LSEC during the fitting process. These highly variable genes were clustered into four different clusters. Among our key genes of interest, JUNB—identified as a highly variable gene along the pseudotime trajectory—was grouped into the fourth cluster (**Figure 12B**). To further explore the role of JUNB in pseudotime progression, through BEAM (Branched Expression Analysis Modeling) analysis, we identified the potential regulatory genes that regulate the branches of LSEC in the pseudo-temporal process, and clustered these genes into four different clusters according to different expression patterns. In BEAM analysis, JUNB was also clustered as a highly variable gene in the first cluster (**Figure 12C**). These results suggest that JUNB may participate in critical regulatory processes during pseudotime. To investigate further, we performed KEGG enrichment analysis on genes in the first cluster (containing JUNB) from BEAM analysis. Consequently, we enriched pathways potentially relevant to LSEC, including “Fluid shear stress and atherosclerosis” and “Leukocyte transendothelial migration” signaling pathways, indicating that LSEC alterations during MAFLD progression may relate to fluid shear stress and leukocyte transendothelial migration (**Supplementary Figure 5G**, **Supplementary Table S4**).

### 3.14 Cell–cell communication

**Figure 13:**
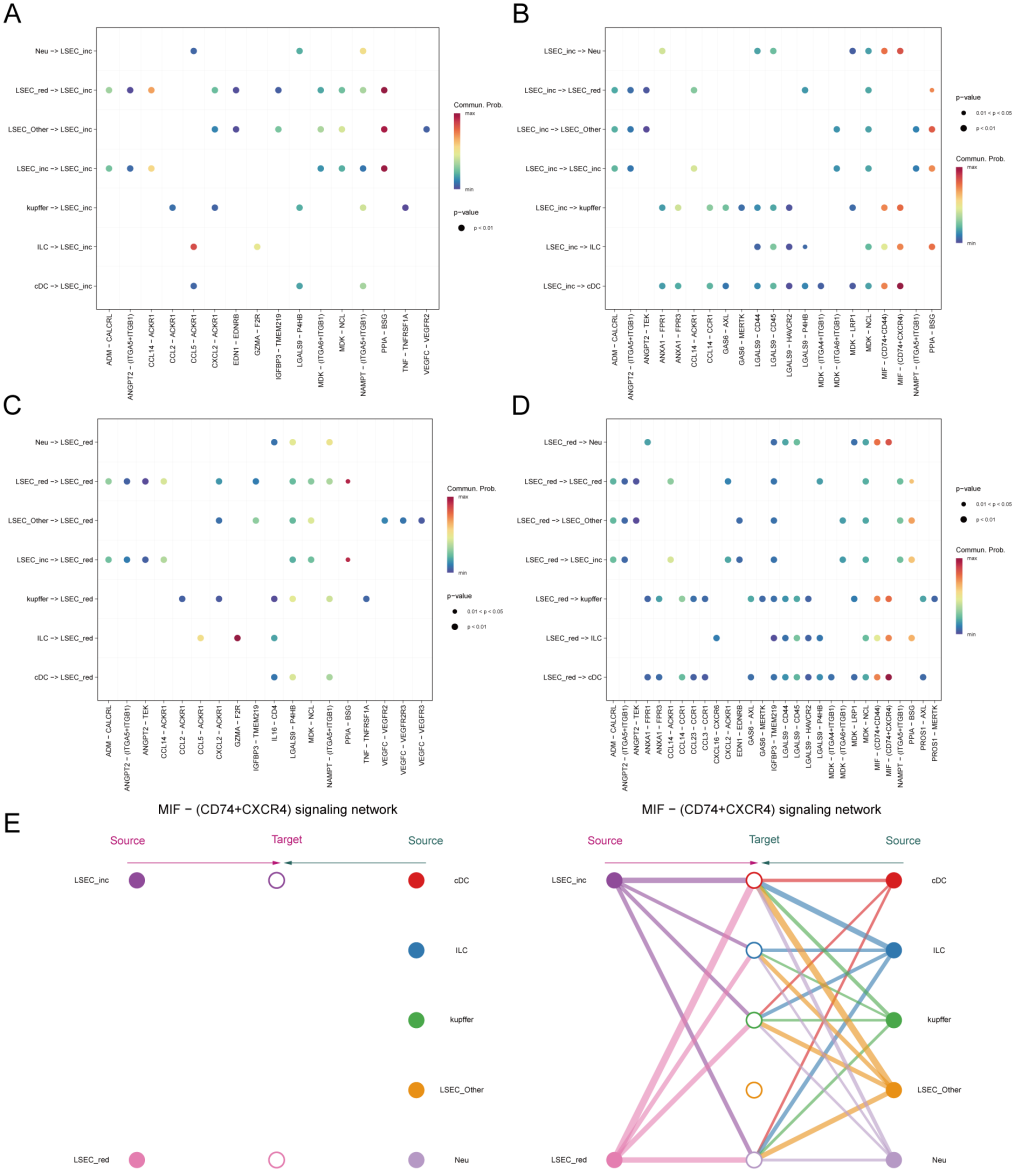
Inferred signaling pathways. **(A, B)** Communication with other cells and the ligand-receptor pairs involved when MAFLD_LSEC_inc act as signal senders and signal receivers, respectively. **(C, D)** Communication with other cells and the ligand-receptor pairs involved when MAFLD_LSEC_red act as signal senders and signal receivers, respectively. **(E)** Hierarchical cell-cell communication plot for ligand-receptor pair “MIF-(CD74+CXCR4)”.

We employed the CellChat package to analyze cell-cell communication networks among five key cell types in MAFLD: LSEC, ILC, Neu, cDC, and kupffer. “LSEC_red” and “LSEC_inc” were defined as cell types at an equivalent hierarchical level to ILC, Neu, cDC, and kupffer. Circular plots depict interaction quantities (**Supplementary Figure 6A**) and interaction strengths (**Supplementary Figure 6B**) among cellular populations. When “LSEC_inc” acts as a signal receiver, it communicates with multiple cell types through ligand-receptor pairs including CCL5-ACKR1 and PPIA-BSG (**Figure 13A**); when serving as a signal sender, it interacts via MIF-(CD74+CD44), MIF-(CD74+CXCR4), and PPIA-BSG (**Figure 13B**). For “LSEC_red” as a receiver, communications occur through GZMA-F2R and PPIA-BSG pairs (**Figure 13C**); as a sender, interactions involve MIF-(CD74+CD44), MIF-(CD74+CXCR4), and PPIA-BSG pairs (**Figure 13D**). The dominant communicating ligand-receptor pairs remained consistently identified, with corresponding signaling pathways classified as: MIF-(CD74+CD44) and MIF-(CD74+CXCR4) belong to the MIF signaling pathway; CCL5-ACKR1 belongs to the CCL signaling pathway; GZMA-F2R belongs to the PARs signaling pathway; PPIA-BSG belongs to the CypA signaling pathway (**Supplementary Tables S5**, **S6**). Consequently, this study focuses on the aforementioned four signaling pathways.

**Figure 14:**
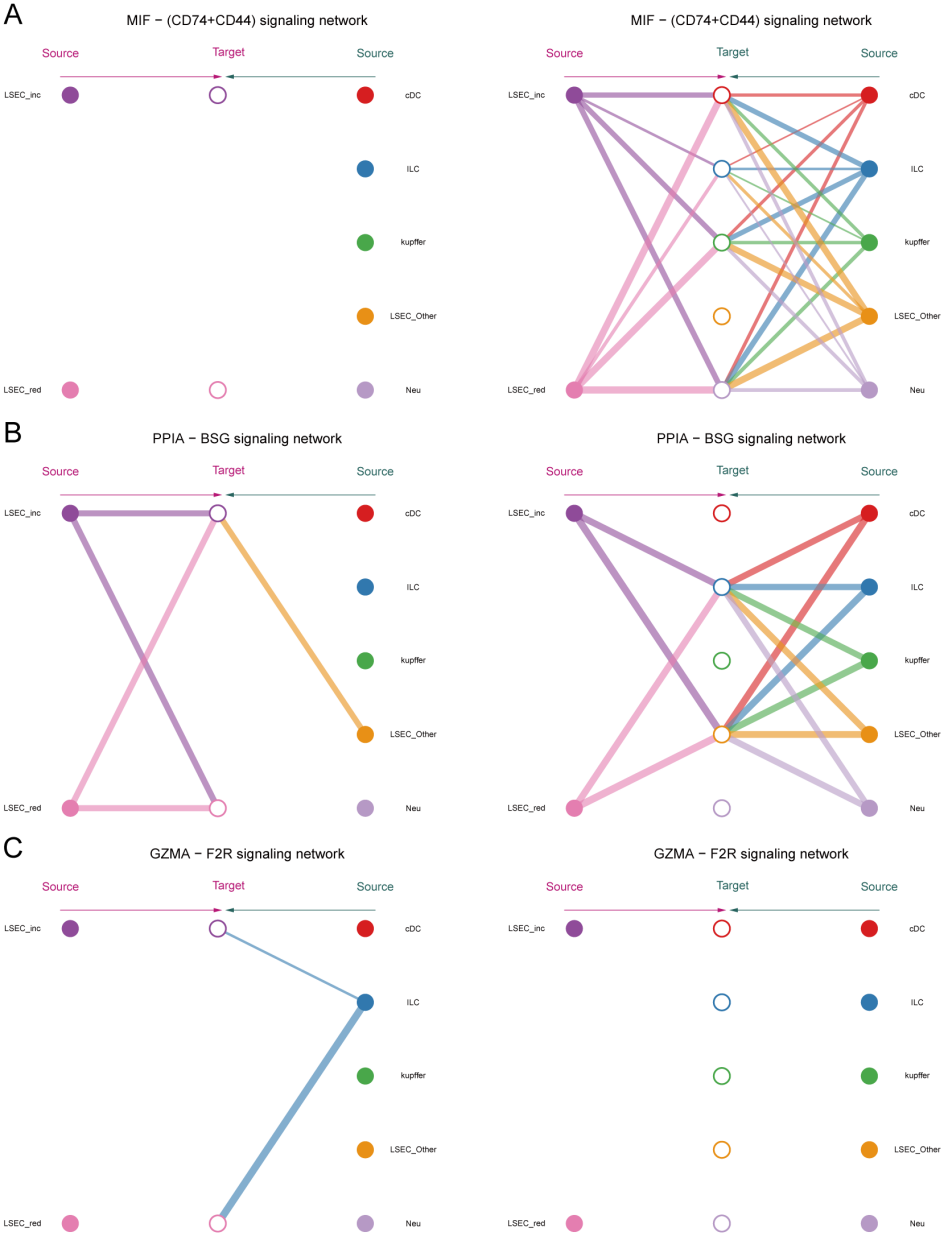
Hierarchical diagrams. **(A–C)** Cellular communication under three distinct ligand-receptor pairs: “MIF-(CD74+CD44)”, “PPIA-BSG”, “GZMA-F2R”.

**Figure 15:**
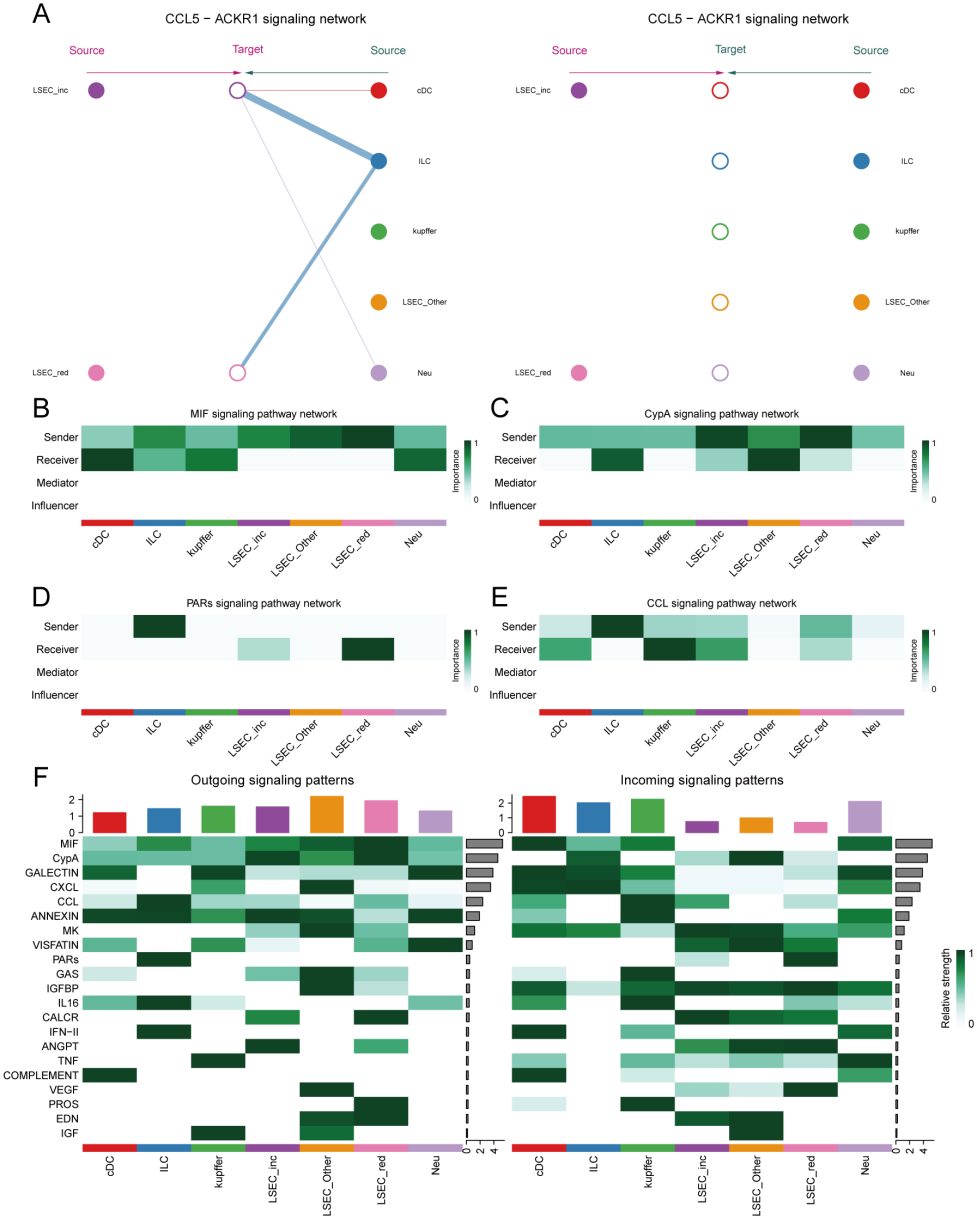
Network centrality scores. **(A)** Hierarchical cell-cell communication plot for ligand-receptor pair “CCL5-ACKR1”. **(B–E)** Network centrality analysis of different cells in the four pathways of MIF, CypA, PARs, and CCL. **(F)** Summary analysis of the possible roles of different cells in the whole communication network.

Within the MIF signaling pathway, both “LSEC_inc” and “LSEC_red” act exclusively as signal senders when communicating with other cells, displaying no self-communication. In hierarchical diagrams corresponding to MIF-(CD74+CD44) and MIF-(CD74+CXCR4), extensive communications exist between LSEC and ILC, Neu, cDC, and kupffer (**Figures 13E**, **14A**). In the CypA pathway, both “LSEC_inc” and “LSEC_red” communicate with kupffer cells as signal senders (**Figure 14B**). Conversely, within the PARs signaling pathway, nearly exclusive communication occurs where “LSEC_red” acts as a signal receiver interacting with ILC (**Figure 14C**). The CCL5 signaling pathway exhibits an inverse pattern, with “LSEC_inc” primarily functioning as a signal receiver communicating with ILC (**Figure 15A**). Subsequently, we ranked ligand-receptor pair contributions across these four pathways (**Supplementary Figures 6D–G**) and consolidated the results. Compared to other ligand-receptor pairs, two pairs from the MIF pathway—”MIF-(CD74+CXCR4)” and “MIF-(CD74+CD44)”—along with the PPIA-BSG pair from the CypA pathway demonstrated significantly higher pathway contributions (**Supplementary Figure 6C**). This highlights the critical importance of these two signaling pathways and the three key ligand-receptor pairs in mediating communications between LSEC and ILC, Neu, cDC, and kupffer.

Finally, by computing network centrality scores, we identified primary signaling roles of distinct cells within the specified pathways mentioned above (**Figures 15B–E**, **Supplementary Figure 7**). Additionally, analysis of signaling roles across all aggregated cell-cell communication networks revealed comprehensively assessed communication identities for different cell types. Both MIF and CypA signaling pathways exhibited the highest activity among all pathways, which aligned with our pathway contribution ranking (**Figure 15F**).

## 4 Discussion

Type 2 diabetes mellitus (T2DM) and metabolic-associated fatty liver disease (MAFLD) are two prevalent metabolic disorders that often co-occur, significantly impacting global health. NAFLD is significantly more prevalent among individuals with T2DM, with some studies indicating that up to 70% of patients with T2DM may also have NAFLD (42). This co-morbidity correlates with a heightened risk of cardiovascular disease, cirrhosis, and hepatocellular carcinoma, contributing to increased morbidity and mortality associated with NAFLD (43). Despite numerous diagnostic and therapeutic strategies, managing T2DM alongside NAFLD remains challenging due to their shared risk factors, including obesity, insulin resistance, and dyslipidemia (44). Furthermore, recent evidence suggests that autophagy plays a significant role in immune response (45), protein secretion, and cellular metabolism (46). It is also crucial in neurological systemic diseases, neuroinflammation, and stroke (47). The pathogenesis of T2DM and MAFLD, along with autophagy dysfunction, is intricately linked; however, the underlying mechanisms remain poorly understood. This study aimed to explore the molecular mechanisms underlying the interplay between T2DM and MAFLD while identifying potential diagnostic biomarkers. We employed an integrated approach comprising differential gene expression analysis, weighted gene co-expression network analysis (WGCNA), protein-protein interaction (PPI) network analysis, and various machine learning techniques to identify key genes and pathways involved in the pathogenesis of these diseases. Additionally, we evaluated the diagnostic accuracy of the identified biomarkers through ROC curve analysis and explored their functional roles using enrichment analysis. Bulk RNA analysis of 98 samples from three datasets (GSE15653, GSE89632, and GSE24807) revealed four genes—CX3CR1, IRAK3, JUNB, and TNFRSF1A—as diagnostic biomarkers associated with autophagy and endoplasmic reticulum stress in T2DM and MAFLD.

CX3CR1, a G protein-coupled receptor (GPCR), is extensively expressed in the human body, particularly in monocytes and macrophages, regulating cellular migration and activation by interacting with its ligand, CX3CL1. It plays a crucial role in various physiological processes and is implicated in significant human diseases like atherosclerosis, rheumatoid arthritis, neurodegenerative diseases, and cancer (48–51), rendering it a promising therapeutic target. For example, activation of CX3CR1 enhances macrophage response to cytokines such as TNF-α and IL-6, which exacerbates liver inflammation and injury, while CX3CR1 is also involved in regulating the migration of immune cells and promotes their aggregation to the liver, which further exacerbates the pathological process of MAFLD (52, 53). In diabetes mellitus combined with metabolic-associated fatty liver disease (MAFLD), aberrant expression and function of CX3CR1 might relate to inflammatory responses and impaired lipid metabolism. Research indicates that CX3CR1 deficiency or dysfunction could exacerbate liver damage associated with diabetes, underscoring its significant contributory role in the development of diabetes combined with MAFLD (54, 55).

Our recent experimental findings indicate a notably elevated expression level of CX3CR1 in the T2DM combined MAFLD group compared to the control group in RT-PCR and immunohistochemistry experiments. This aligns with previous research outcomes. Furthermore, the CX3CR1 signaling pathway is intricately linked to the generation of various cytokines, offering novel perspectives for potential therapeutic interventions in the context of diabetes combined with MAFLD. Recent literature has suggested that cholesterol regulates the activation of CX3CR1 (56). Additionally, function of CX3CR1 in MAFLD may confused now, as CX3CR1 knockout mice exhibit increased susceptibility to high-fat diet-induced obesity, insulin resistance, hepatic degeneration, and inflammation (57). Research indicates that CX3CR1 significantly regulates autophagy. Its activation suppresses the autophagic process in macrophages, causing intracellular metabolite accumulation and cellular dysfunction, notably pronounced in diabetes with MAFLD (58). Specifically, the CX3CL1-CX3CR1 signaling pathway downregulates autophagy-related gene expression through key regulators like CaMKIIδ and HDAC4, impacting macrophage survival and function (59). Single-cell communication analyses revealed that MAFLD-associated LSEC and various other cells engage in intercellular communication via the macrophage migration inhibitory factor (MIF) pathway. Hepatocytes and Kupffer cells are identified as primary sources of hepatic MIF (60). Our analysis identified that the intercellular communication within the MIF signaling pathway predominantly relies on the receptor-ligand pair “CD74-CXCR4,” with the ligand CXCR4 belonging to the same “chemokine receptor family” as CX3CR1, which is a potential biomarker for MAFLD identified in bulk analyses (61). These findings imply a potential synergistic effect between the CX3CL1-CX3CR1 signaling pathway and the MIF pathway, particularly concerning the role of LSEC. Further investigation is warranted to explore any deeper connections between these pathways and their relation to MAFLD development. Furthermore, autophagy inhibition is closely linked to diabetes-related inflammatory responses, subsequently enhancing hepatic fat accumulation and fibrosis (62). Nonetheless, the pathophysiological role of the CX3CL1-CX3CR1 signaling pathway in NAFLD development is still debated. In mouse liver, CX3CL1 is expressed in Kupffer cells, hepatic macrophages, and hematopoietic stem cells (21), while CX3CR1 primarily localizes to Kupffer cells (63).Kupffer cells are resident macrophages in the liver. Studies indicate that the interaction between CX3CL1 and CX3CR1 suppresses the inflammatory properties of Kupffer cells/macrophages, thereby alleviating hepatic inflammation and fibrosis (63).Studies have shown that in the absence of CX3CR1, hepatic monocytes preferentially differentiate into macrophages that produce pro-inflammatory tumor necrosis factor and inducible nitric oxide synthase. CX3CR1 serves as a critical survival signal for monocyte-derived macrophages in the liver by activating the expression of the anti-apoptotic protein Bcl-2. Monocytes/macrophages lacking CX3CR1 exhibit increased cell death following liver injury, thereby sustaining inflammation, promoting persistent recruitment of inflammatory monocytes to the liver, and exacerbating hepatic fibrosis. Thus, CX3CR1 limits liver fibrosis *in vivo* by regulating the differentiation and survival of intrahepatic monocytes (64).Studies have also shown that administration of CX3CR1 agonists (such as CXCL16 analogs) can reduce Kupffer cell activation, decrease TNF-α/IL-6 levels, and ameliorate steatohepatitis in high-fat diet mouse models (65).In our study, through single-cell analysis, we discovered CX3CR1 predominantly enriched in neutrophils and innate lymphoid cells. Kupffer cells communicate with neutrophils and innate lymphoid cells via the MIF-(CD74+CXCR4) ligand-receptor pair in the MIF signaling pathway. Thus, we speculate that the enrichment state of CX3CR1 in non-alcoholic steatohepatitis may undergo alterations during disease progression. It should be noted that this dynamic pattern requires further validation through future research.

Experimental results indicated significantly elevated IRAK3 expression in the T2DM and MAFLD group compared to the control group, suggesting that IRAK3 may play a vital role in the pathophysiology of T2DM and MAFLD. IRAK3 has been shown to regulate inflammatory responses effectively. As a negative regulator, IRAK3 curtails excessive inflammatory responses by inhibiting key molecules in downstream signaling pathways. For instance, IRAK3 regulates endogenous immune signaling via its guanylate cyclase activity, thereby inhibiting the production of inflammatory cytokines like tumor necrosis factor-α (TNF-α) and interleukin-6 (IL-6) (66). In models of disease, such as acute pancreatitis, IRAK3 deficiency results in heightened inflammation, underscoring its importance in maintaining immune homeostasis (67). Additionally, IRAK3 inhibits the activation of the NF-κB pathway through interaction with the MyD88 pathway, thereby mitigating the severity of the inflammatory response (68). Consequently, IRAK3 functional deficiency may contribute to the emergence of multiple inflammation-related diseases, highlighting its potential as a therapeutic target. Furthermore, IRAK3 regulates intrinsic immune signaling through its guanylate cyclase activity, further influencing the release of inflammatory mediators. The regulatory mechanism by which IRAK3 influences autophagy is receiving increasing attention. Autophagy, a cellular self-degradation process, is essential for maintaining intracellular homeostasis and managing stress responses. Research indicates that IRAK3 is not only involved in regulating inflammatory signaling but also influences autophagy activity. For instance, IRAK3 expression levels were negatively correlated with autophagy-related genes, indicating that IRAK3 may modulate cell survival and death by inhibiting autophagy (68). Moreover, IRAK3 affects the autophagic process by influencing intracellular cGMP levels; an increase in cGMP promotes autophagy, counteracting IRAK3’s inhibitory effect to some extent (69). Thus, IRAK3’s role in regulating autophagy offers new insights into its dual functionality in inflammation and cell fate decisions. In this study, bioinformatics analysis demonstrated IRAK3’s association with the IL1R pathway and TLR1 and TLR2 cascades, wherein IRAK3 expression diminishes downstream signaling by inhibiting the activation of IRAK1, subsequently reducing the production of inflammatory factors (70). Additionally, IRAK3 is intricately linked to the cascade responses of TLR1 and TLR2, regulating TLR-mediated immune responses and affecting the activation status of macrophages and monocytes (71). In the context of infection and inflammation, IRAK3 prevents immune overreaction by negatively regulating the TLR signaling pathway, thus safeguarding host tissues from damage. This mechanism has been validated across various disease models, underscoring the complexity and significance of IRAK3 in regulating immune responses (72). Furthermore, IRAK3 introduces additional regulation by producing cyclic guanosine monophosphate (cGMP) through its guanylate cyclase activity. cGMP inhibits NF-κB activity and reduces cytokine levels in response to inflammatory stimuli. This cGMP-mediated pathway may represent a novel mechanism by which IRAK3 exerts its anti-inflammatory effects, providing new therapeutic avenues for managing inflammatory responses in T2DM and MAFLD (66, 73).

JUNB, a significant transcription factor, is engaged in diverse biological processes such as cell proliferation, differentiation, and apoptosis. Our present experimental validation indicated markedly elevated JUNB expression in the T2DM combined with metabolic-associated fatty liver disease (MAFLD) group compared to the control group. Research indicates a direct correlation between JUNB expression levels and metabolic disorders, impacting fat metabolism, insulin sensitivity, and inflammatory reactions. For instance, JUNB deficiency boosts adipocytes’ calorie consumption, ameliorating diet-induced insulin resistance, implying an inhibitory function of JUNB in energy metabolism (74). Furthermore, JUNB significantly influences hepatic metabolism and might contribute to the pathogenesis of nonalcoholic fatty liver disease by modulating lipid synthesis and catabolism in hepatocytes (75). Our single-cell analysis unearthed an additional mechanism impacting NAFLD through metabolic pathways. Through the identification of core mutated genes in differentiation trajectories, we observed the pivotal role of the “Lipid and atherosclerosis” pathway in LSEC differentiation and development. This pathway is intricate, involving lipid metabolism, vascular biology, and cardiovascular diseases. Maintaining hepatic cholesterol homeostasis is critical for hepatic function. Disruptions in this balance can result in MAFLD and atherosclerotic cardiovascular disease (76).Moreover, BEAM analysis revealed the critical “Leukocyte transendothelial migration” pathway associated with LSEC differentiation. Our findings are substantiated by evidence demonstrating that LSECs from MAFLD patients exhibit enhanced leukocyte migratory capacity (77). Liver inflammation is recognized as a hallmark of MAFLD progression, with multiple studies indicating immune cell remodeling during this process correlates with disease severity and hepatic fibrosis (78, 79). In conclusion, JUNB plays essential roles in modulating inflammation through diverse mechanisms, maintaining immune homeostasis, and preventing excessive inflammation. Given the chronic inflammation associated with T2DM and MAFLD, targeting JUNB may offer novel therapeutic interventions for these conditions.

Bioinformatics analysis indicated that JUNB is predominantly enriched in the TNF signaling pathway, TGF-beta signaling pathway, interleukin 4 and interleukin 13 signaling, IL6, and IL7 pathways, playing a pivotal role in various cellular signaling pathways, particularly in inflammation and metabolic regulation. The TNF signaling pathway regulates inflammation by modulating inflammatory responses, with JUNB regulating the expression of TNF-related genes (80). Additionally, JUNB exerts a crucial influence in the TGF-beta signaling pathway, impacting cell proliferation and fibrosis, closely associated with the development of metabolic disorders (80). JUNB’s involvement in the interleukin 4 and interleukin 13 signaling pathways underscores their significance in immune responses and metabolic processes (81). Similarly, JUNB’s connection to the IL-6 and IL-7 pathways highlights the direct link between overexpression of IL-6 and metabolic syndrome development (82), suggesting JUNB’s role in metabolic status regulation through IL-6 signaling modulation. Hence, targeting JUNB in these signaling pathways presents a promising therapeutic strategy for metabolic diseases, aligning with our experimental findings. The emerging recognition of JUNB’s role in the autophagy mechanism is gaining prominence, with studies demonstrating JUNB’s ability to modulate cellular autophagy levels by regulating autophagy-related gene expression. Notably, JUNB’s overexpression correlates with autophagy inhibition, as evidenced in an osteoarthritis model, indicating a potential promotion of articular cartilage degradation by JUNB through autophagy suppression (83).Recent studies have indicated that upregulation of JUNB impairs hepatic lipid metabolism in mice, while inhibition of JUNB can alleviate lipid accumulation in the liver (84). Additionally, research in lymphoma suggests that inhibiting mTOR can downregulate JunB protein levels and reduce cell proliferation, indicating that JunB is a crucial target of mTOR (85). However, the mechanisms by which JUNB modulates the mTOR signaling pathway in the context of T2DM and MAFLD remain unclear.

TNFRSF1A, a vital cell membrane receptor, crucially regulates cell survival, proliferation, and apoptosis. Our study revealed a notable upregulation of TNFRSF1A in T2DM with MAFLD, aligning with earlier research. The heightened TNFRSF1A expression correlated closely with insulin resistance, inflammation, and hepatic lipid deposition. Recent studies have shown that immune dysregulation is observed in MAFLD. TNFRSF1A is closely related to immune regulation. TNFRSF1A can improve the sensitivity and accuracy of diagnosing and predicting T2DM-related MAFLD, thus providing new diagnostic options for patients with T2DM and MAFLD (86).

Notably, bioinformatics analysis identified TNFRSF1A enrichment in the TNF, MAPK, and NF-kappa B signaling pathways. TNFRSF1A, a pivotal player in the TNF pathway, orchestrates cellular responses to TNF-α. By stimulating NF-kappa B and MAPK pathways, TNFRSF1A drives inflammation and apoptosis. In the diabetic MAFLD context, elevated TNF-α levels trigger TNFRSF1A and subsequent NF-kappa B activation, leading to downstream inflammatory mediator expression, exacerbating liver damage and metabolic irregularities. Furthermore, MAPK pathway activation influences cell dynamics like proliferation, apoptosis, and inflammation, providing fresh insights into diabetes with MAFLD mechanisms (87, 88).

Furthermore, TNFRSF1A is intricately linked with the NF-kappa B and MAPK signaling pathways through its endogenous structural domains, thereby establishing a complex signaling network. In diabetes with MAFLD, TNFRSF1A activation induces NF-kappa B translocation, resulting in the production of pro-inflammatory factors like IL-6 and TNF-α, which exacerbate liver inflammation. Additionally, TNFRSF1A enhances cellular stress responses and apoptosis via MAPK signaling pathway activation, contributing to hepatocyte injury and fat accumulation. Activated endothelial cells in the liver facilitate the subendothelial migration of monocytes, which can further differentiate into macrophage-like cells or mobile dendritic cells, accumulating in hepatic tissue. Chemotactic stimulation enhances cellular recruitment, while the inhibition of JAK-STAT signaling can reduce monocyte migration (89). In our GSEA enrichment analysis of potential MAFLD marker genes, we identified proliferation in the KEGG_CYTOKINE_CYTOKINE_RECEPTOR_INTERACTION pathway linked to the JAK-STAT pathway, emphasizing that TNFRSF1A significantly promotes inflammatory responses. Consequently, TNFRSF1A serves as a crucial biomarker for diabetes in conjunction with MAFLD and presents as a promising therapeutic target (90). Our findings revealed that TNFRSF1A and JUNB are key genes enriched within the TNF signaling pathway, essential for elucidating the molecular mechanisms underlying T2DM and MAFLD. TNFRSF1A encodes TNFR1, a mediator of pro-inflammatory responses and apoptosis pivotal in pathogenesis of insulin resistance and liver inflammation (91). JUNB, a component of the AP-1 transcription factor, regulates gene expression in response to TNF signaling and significantly contributes to the inflammatory process. The enrichment of TNFRSF1A and JUNB within the TNF signaling pathway indicates their potential as diagnostic biomarkers for T2DM coupled with MAFLD. Their roles in critical inflammatory and apoptotic pathways highlight the significance of targeting TNF signaling to mitigate disease progression. Furthermore, therapeutic interventions aimed at modulating TNF signaling may concurrently address both metabolic and hepatic dysfunction in affected patients (92).

Finally, our single-cell analysis also has some methodological limitations: while our computational approach substantially mitigated batch effects inherent in bulk RNA-seq, it lacks complementary single-cell expression profiling or *in situ* validation. This limitation precludes direct evidence of how the signature genes (CX3CR1, IRAK3, JUNB, TNFRSF1A) dynamically modulate LSEC functionality during MAFLD progression.

## 5 Conclusion

In conclusion, this study provides critical insights into the molecular mechanisms underlying the comorbidity of T2DM and MAFLD. Utilizing comprehensive bioinformatics approaches, we identified multiple differentially expressed genes and pivotal signaling pathways that potentially mediate the co-progression of T2DM and MAFLD. However, this study has several limitations. The primary constraint lies in the relatively small sample size of human liver biopsy datasets meeting both T2DM and MAFLD phenotypic criteria in GEO databases (GSE15653, n=14; GSE23343, n=17), which may reduce statistical power for detecting subtle molecular alterations and limit the generalizability of findings across diverse populations. Although these datasets underwent rigorous quality control, the scarcity of publicly available samples fulfilling dual diagnostic criteria remains a significant challenge for contemporary bioinformatics research in this field. To address these issues, we are implementing proactive monitoring of newly released public datasets and initiating multicenter collaborations to expand sample collections, thereby supporting subsequent validation studies.

## Data Availability

The datasets generated and/or analysed during the current study are available in the NCBI repository, and the data used to support the findings of this study have been deposited in the GEO repository (GSE15653, GSE89632, and GSE24807 and GSE136103).

## Supplementary material

Supplementary Figure 1 Flow diagram of this study.

Supplementary Figure 2
**(A, E)** Sample dendrogram (after removing outlier samples) and feature heatmap.**(B, F)** Clustering dendrogram. **(C, G)** Clustering dendrogram of modules. **(D, H)** Inter-module correlation heatmap.

Supplementary Figure 3 GSEA (Gene Set Enrichment Analysis).

Supplementary Figure 4 Correlation between genes and immune cells. IRAK3 **(A)**; TNFRSF1A **(B–G)**; CX3CR1 **(H)**; JUNB **(I)**. Correlation network heatmap **(J)**.

Supplementary Figure 5 Pseudo-temporal trajectory analysis on LSEC. **(A)** Dendrogram at 0.6 resolution. **(B)** Staged trajectory plot. **(C)** Trajectory plot of distinct cell sub-populations. **(D)** Pseudo-time direction. **(E)** The cell density plot along the timeline. **(F)** Distribution differences of ECs across groups. **(G)** KEGG enrichment analysis for the gene cluster containing JUNB.

Supplementary Figure 6 Cell–cell communication. **(A, B)** Number of interactions between cell populations and their strength. **(C–G)** Comparison of the ranking of the contribution of all ligand receptor pairs of the four pathways MIF, CypA, PARs, CCL.

Supplementary Figure 7 Network centrality scores. **(A–E)** Communication identities of different cells in specific pathways (MIF, CypA, PARs, CCL) and integrated communication identities.

Supplementary Table 1 A summary of Microarray Information.

Supplementary Table 2 List of Primers.

Supplementary Table 3 The expression levels of various factors between the T2DM with MAFLD group and the Control group.

Supplementary Table 4 List of KEGG.

Supplementary Table 5 List of ligand-receptor pairs (source).

Supplementary Table 6 List of ligand-receptor pairs (target).

Supplementary File 1 KEGG enrichment analysis results of WGCNA core module genes in the GSE89632_SS group.

Supplementary File 2 KEGG enrichment analysis results of WGCNA core module genes in the GSE89632_NASH group.
