# Supplementary figures and images for: Exploring the biological functions and immune regulatory roles of IRAK3, TNFRSF1A, CX3CR1, and JUNB in T2DM combined with MAFLD: integrated bioinformatics and single-cell analysis
Source: Front Immunol. 2025 Aug 22;16:1587225. doi: 10.3389/fimmu.2025.1587225 (PMC12411428; doi:10.3389/fimmu.2025.1587225)

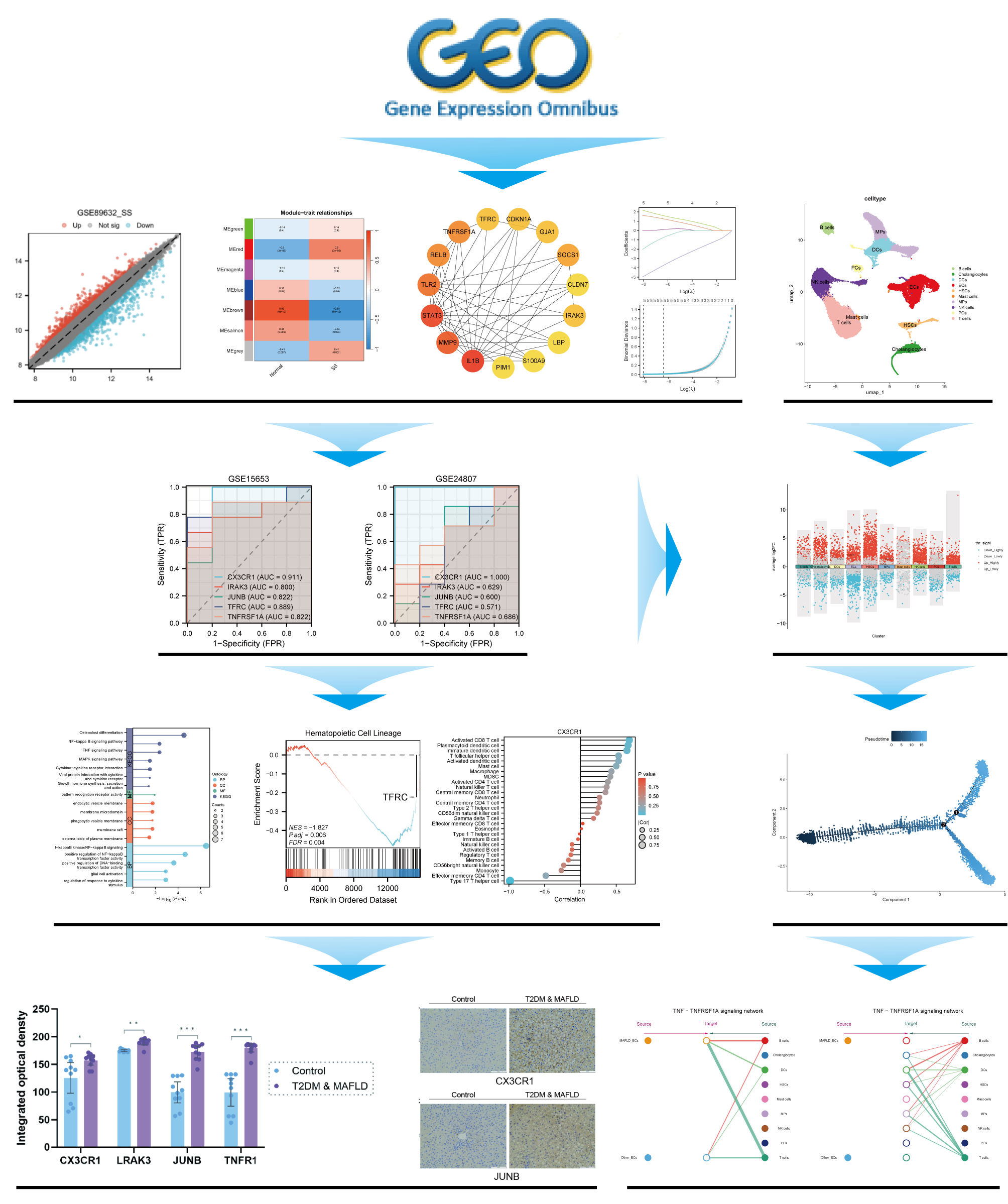

Supplement: Supplementary Figure 1 — Flow diagram of this study. [file Image1.tif]

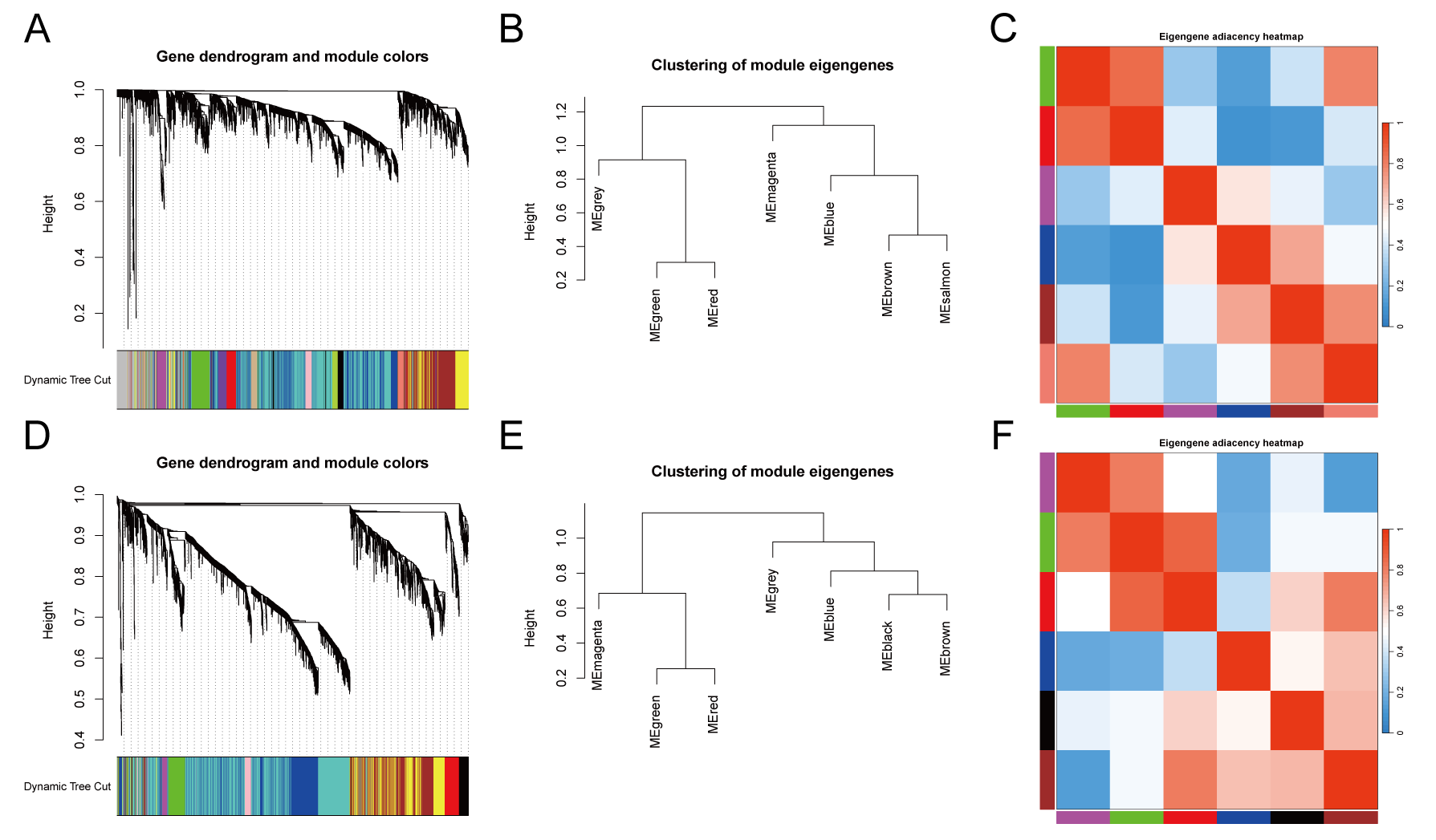

Supplement: Supplementary Figure 2 — (A, E) Sample dendrogram (after removing outlier samples) and feature heatmap.(B, F) Clustering dendrogram. (C, G) Clustering dendrogram of modules. (D, H) Inter-module correlation heatmap. [file Image2.tif]

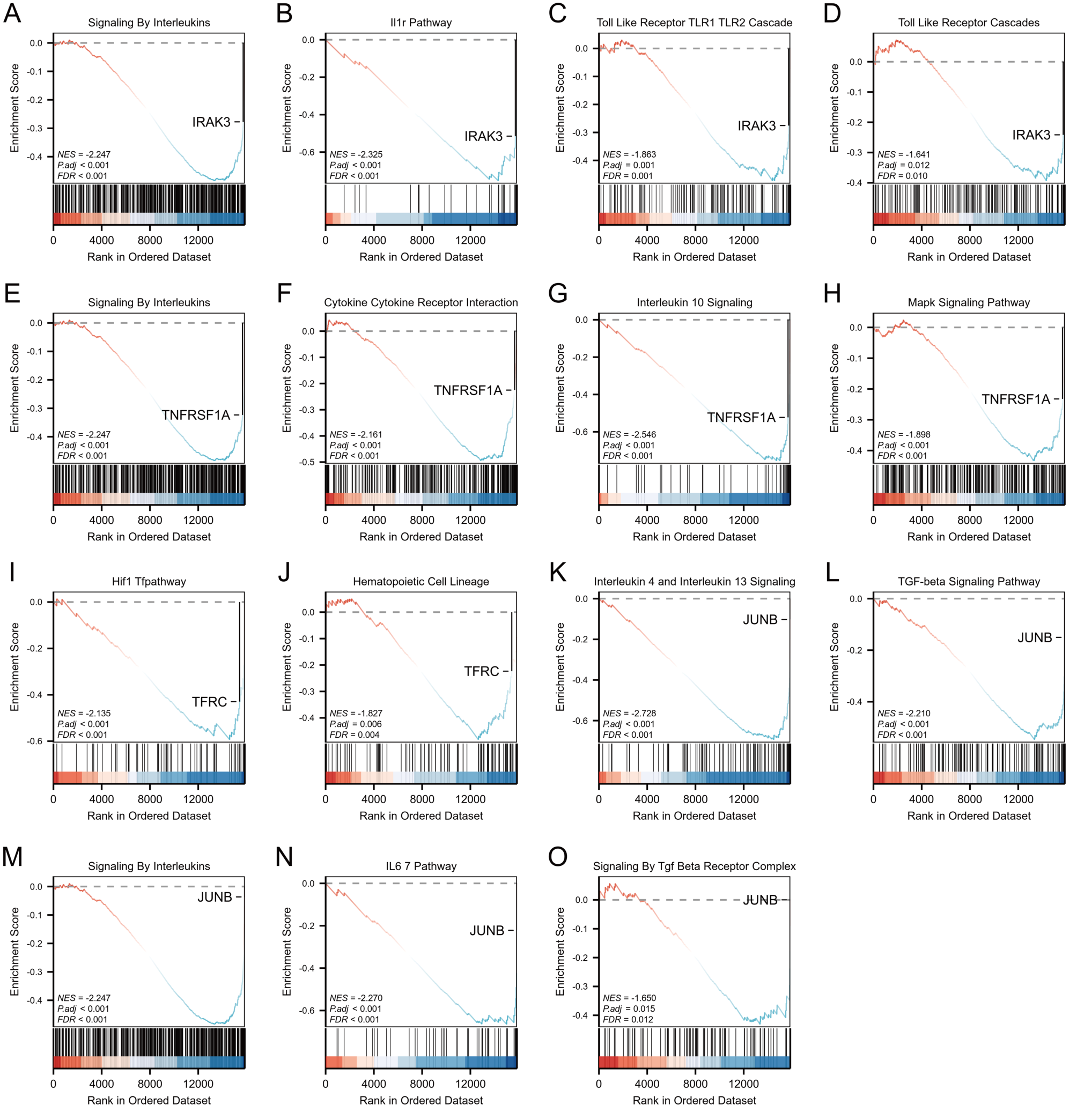

Supplement: Supplementary Figure 3 — GSEA (Gene Set Enrichment Analysis). [file Image3.tif]

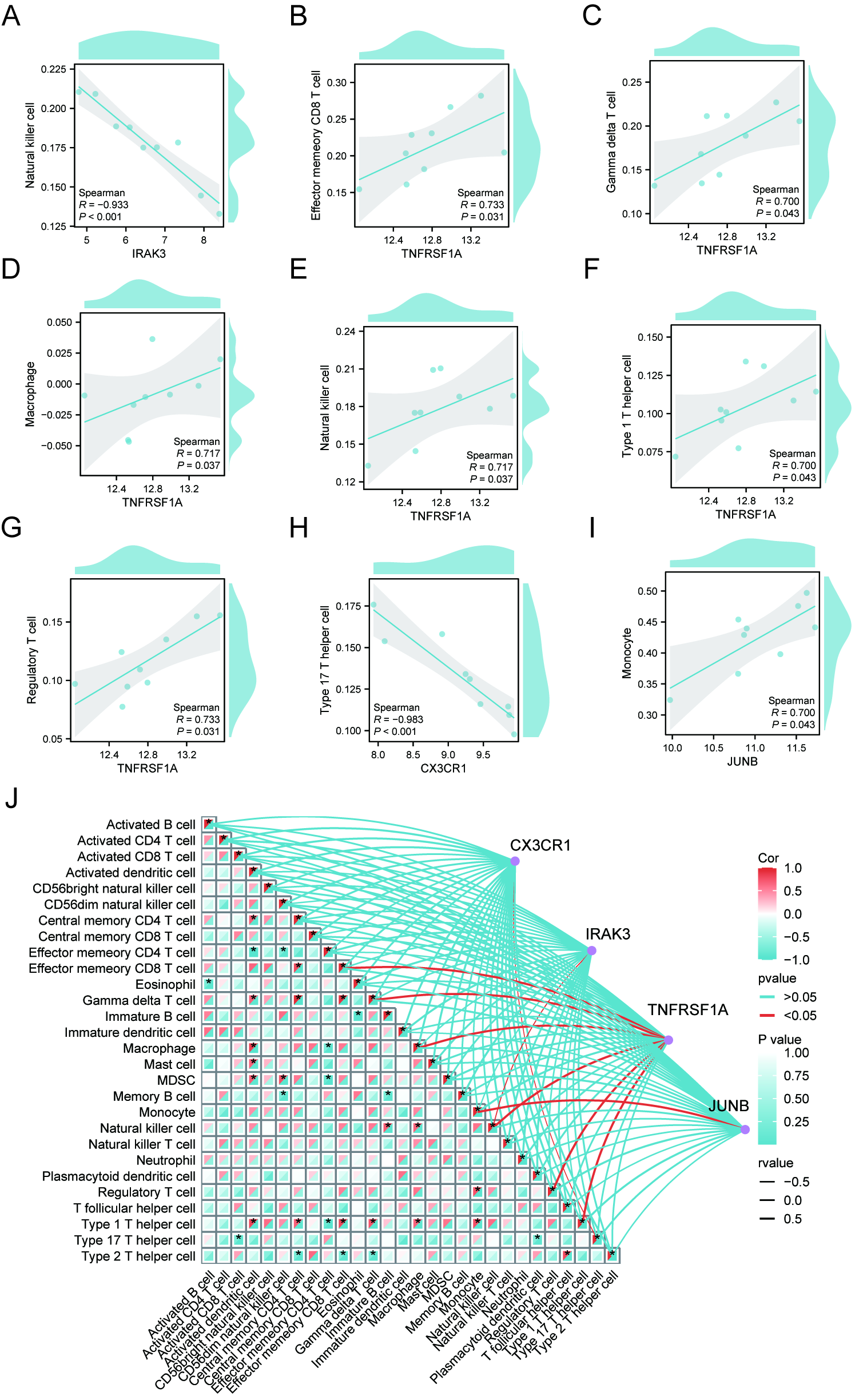

Supplement: Supplementary Figure 4 — Correlation between genes and immune cells. IRAK3 (A); TNFRSF1A (B–G); CX3CR1 (H); JUNB (I). Correlation network heatmap (J). [file Image4.tif]

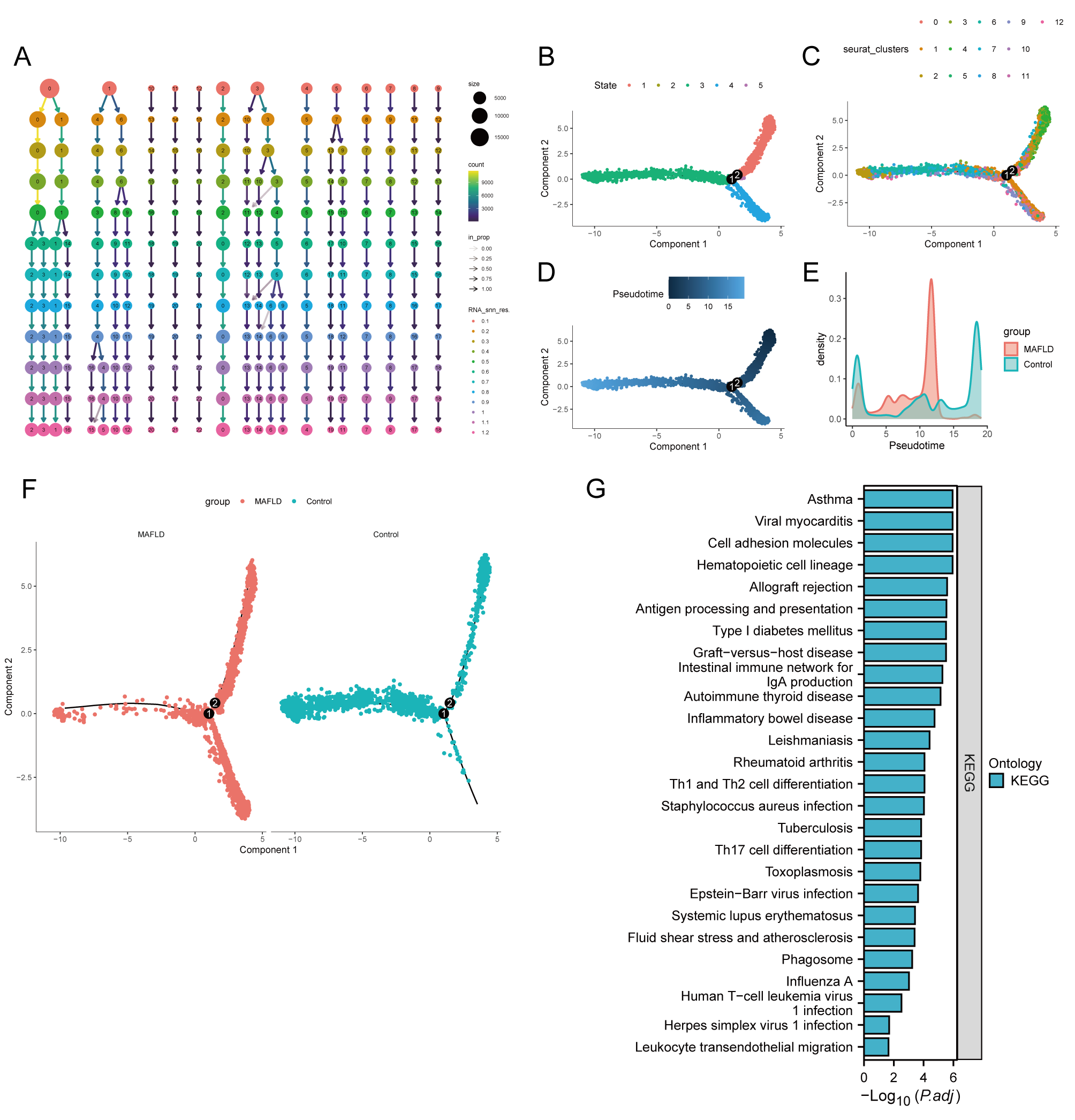

Supplement: Supplementary Figure 5 — Pseudo-temporal trajectory analysis on LSEC. (A) Dendrogram at 0.6 resolution. (B) Staged trajectory plot. (C) Trajectory plot of distinct cell sub-populations. (D) Pseudo-time direction. (E) The cell density plot along the timeline. (F) Distribution differences of ECs across groups. (G) KEGG enrichment analysis for the gene cluster containing JUNB. [file Image5.tif]

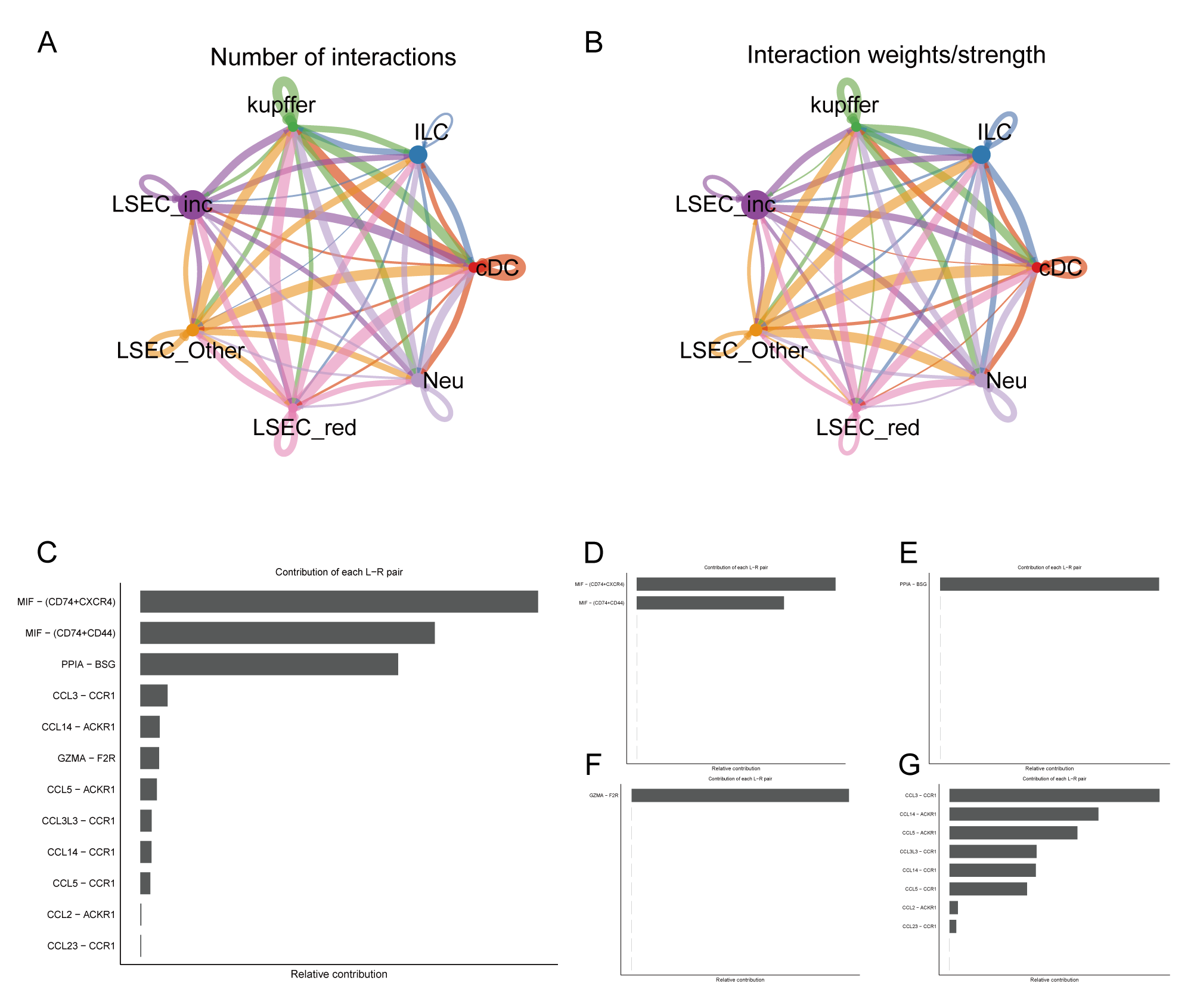

Supplement: Supplementary Figure 6 — Cell–cell communication. (A, B) Number of interactions between cell populations and their strength. (C–G) Comparison of the ranking of the contribution of all ligand receptor pairs of the four pathways MIF, CypA, PARs, CCL. [file Image6.tif]

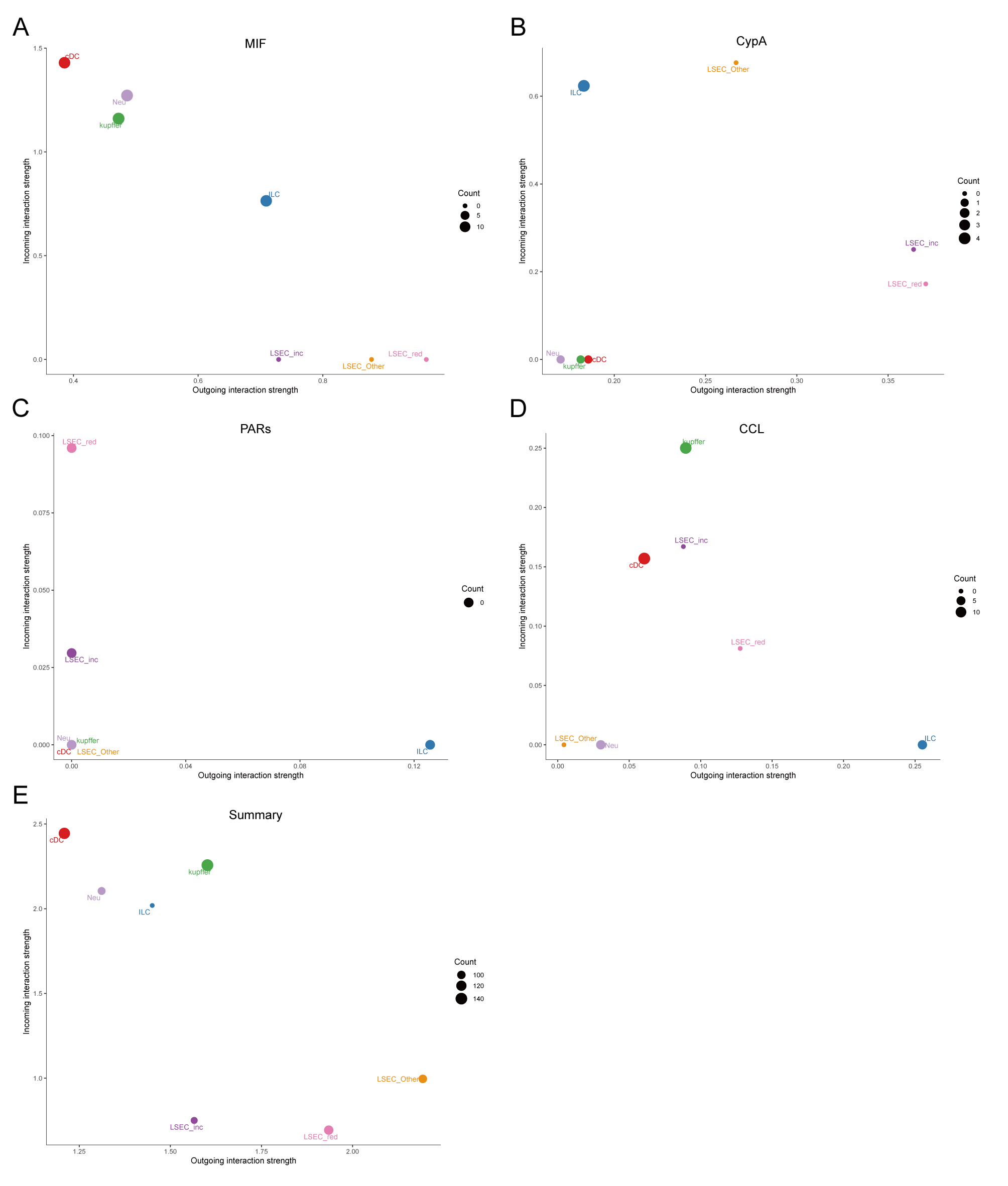

Supplement: Supplementary Figure 7 — Network centrality scores. (A–E) Communication identities of different cells in specific pathways (MIF, CypA, PARs, CCL) and integrated communication identities. [file Image7.tif]
