# Supplementary material for: Exploring the biological functions and immune regulatory roles of IRAK3, TNFRSF1A, CX3CR1, and JUNB in T2DM combined with MAFLD: integrated bioinformatics and single-cell analysis
Source: Front Immunol. 2025 Aug 22;16:1587225. doi: 10.3389/fimmu.2025.1587225 (PMC12411428; doi:10.3389/fimmu.2025.1587225)
Supplement: Supplementary Table 1 — A summary of Microarray Information. [file Table1.docx]

**Table 1** A summary of Microarray Information

| Series | Group | | | | | Tissue | Platfrom |
| --- | --- | --- | --- | --- | --- | --- | --- |
|  | T2DM | T2DM with Obese | NASH | SS | Control |  |  |
| GSE15653 |  | 9 |  |  | 5 | Liver | GPL96 |
| GSE89632_NASH |  |  | 19 |  | 24 | Liver | GPL14951 |
| GSE89632_SS |  |  |  | 20 | 24 | Liver | GPL14951 |
| GSE24807 |  |  | 12 |  | 5 | Liver | GPL2895 |
| GSE23343 | 10 |  |  |  | 7 | Liver | GPL570 |
