# Supplementary material for: Exploring the biological functions and immune regulatory roles of IRAK3, TNFRSF1A, CX3CR1, and JUNB in T2DM combined with MAFLD: integrated bioinformatics and single-cell analysis
Source: Front Immunol. 2025 Aug 22;16:1587225. doi: 10.3389/fimmu.2025.1587225 (PMC12411428; doi:10.3389/fimmu.2025.1587225)
Supplement: Supplementary Table 2 — List of Primers. [file Table2.docx]

**Table 2 List of** **Primers**

| **Primers** | **Sequence** |
| --- | --- |
| GAPDH-F | CAAGTTCAACGGCACAG |
| GAPDH-R | CCAGTAGACTCCACGACAT |
| IRAK3-F | CGATTTCCCAACTTACCA |
| IRAK3-R | AAGTGTCCCATTGCTCAT |
| TNFRSF1A-F | GTACTGCCGTGCTGTTGC |
| TNFRSF1A-R | GCTGAAGGCTGGGATAGAG |
| CX3CR1-F | CCTTTATCTACGCTTTCGC |
| CX3CR1-R | TGCTCCTCTGGGACTCTGT |
| JUNB-F | CTACAAACTCCTGAAACCCACC |
| JUNB-R | TCTGATCCCTGACCCGAAA |
