# Supplementary material for: Exploring the biological functions and immune regulatory roles of IRAK3, TNFRSF1A, CX3CR1, and JUNB in T2DM combined with MAFLD: integrated bioinformatics and single-cell analysis
Source: Front Immunol. 2025 Aug 22;16:1587225. doi: 10.3389/fimmu.2025.1587225 (PMC12411428; doi:10.3389/fimmu.2025.1587225)
Supplement: Supplementary Table 3 — The expression levels of various factors between the T2DM with MAFLD group and the Control group. [file Table3.docx]

**Table 3.** The expression levels of various factors between the T2DM with MAFLD group and the Control group

| Variable (group/N) | Statistic description | Method | Statistics | P value |
| --- | --- | --- | --- | --- |
| CX3CR1 |  | W | 23.00 | 0.045 |
| Control (10) | 139.814(61.031) |  |  |  |
| T2DM & MAFLD (10) | 160.705(12.56) |  |  |  |
| LRAK3 |  | W | 8.00 | 0.002 |
| Control (10) | 175.278(2.241) |  |  |  |
| T2DM & MAFLD (10) | 193.138(6.905) |  |  |  |
| JUNB |  | t | -7.50 | <0.001 |
| Control (10) | 99.504±26.608 |  |  |  |
| T2DM & MAFLD (10) | 172.318±15.334 |  |  |  |
| TNFR1 |  | W | <0.01 | <0.001 |
| Control (10) | 111.431(64.035) |  |  |  |
| T2DM & MAFLD (10) | 184.188(6.205) |  |  |  |
